# Supplementary material for: A novel anti-epileptogenesis strategy of temporal lobe epilepsy based on nitric oxide donor
Source: EMBO Mol Med. 2024 Dec 9;17(1):85–111. doi: 10.1038/s44321-024-00168-1 (PMC11730642; doi:10.1038/s44321-024-00168-1)
Supplement: Supplementary file 1 — Appendix [file 44321_2024_168_MOESM1_ESM.pdf]

Table of contents

File	Page
**Table of contents**	1
**Appendix figures and legends**	2
Appendix Figure S1	2
Appendix Figure S1 legend	2
Appendix Figure S2	3
Appendix Figure S2 legend	3
Appendix Figure S3	4
Appendix Figure S3 legend	4
Appendix Figure S4	5
Appendix Figure S4 legend	5
**Statistics tables**	6
Appendix Table S1	6
Appendix Table S2	8
Appendix Table S3	9
Appendix Table S4	10
Appendix Table S5	11
Appendix Table S6	13
Appendix Table S7	17
Appendix Table S8	19
Appendix Table S9	20
Appendix Table S10	21
Appendix Table S11	22
Appendix Table S12	25
Appendix Table S13	25
Appendix Table S14	25

Appendix figures and legends

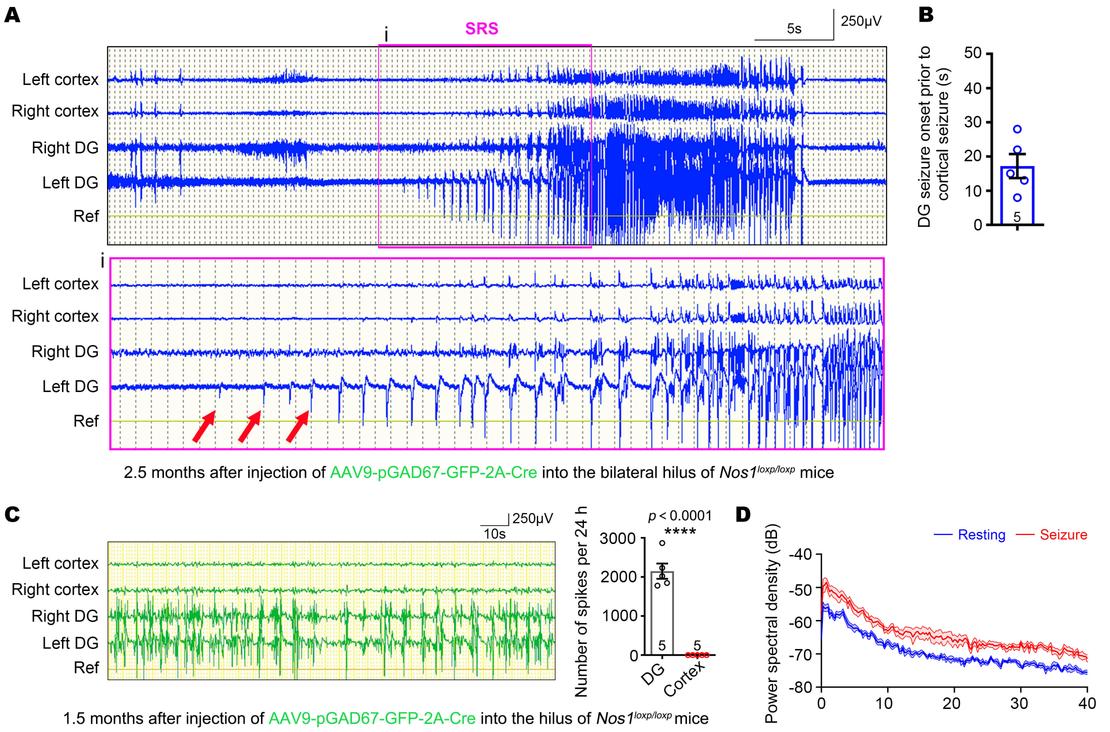

Appendix Figure S1. Epileptogenesis originates from the hippocampal DG after the conditional deletion of nNOS in hilar interneurons.

(**A**) Representative occurrence of an SRS 2.5 months after injection of AAV9-pGAD67-GFP-2A-Cre virus into the bilateral hilus of *Nos1*$^{loxp/loxp}$ mice. Note the spontaneous spikes of an SRS started from the DG. The same phenomenon was observed in 5 mice. (i) was zoomed picture from pink square. (**B**) Data graph showing the onset time of DG SE prior to cortical SE. (**C**) Representative EEG and data graph showing epileptic spikes in DG 1.5 months after injection of AAV9-pGAD67-GFP-2A-Cre into the hilus of *Nos1*$^{loxp/loxp}$ mice. Student's *t*-test, n = 5 mice. (**D**) The power spectrum of EEG recordings in resting and seizure state. Error bars correspond to ± s.e.m. ****$P < 0.0001$, *NS*, not significant.

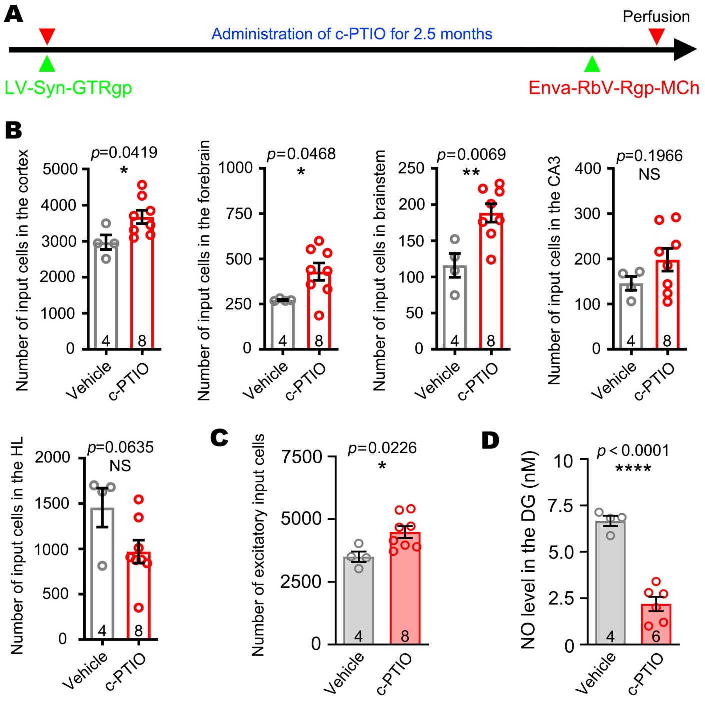

Appendix Figure S2. Clearance of NO leads to the development of hyper-excitation afferent circuits onto DGCs.

(**A**) Experimental paradigm for tracing the input of DGCs after systematic deprivation of NO by cPTIO. (**B**) Data graphs show connectivity ratios of input neurons located in the cortex, forebrain, brainstem, CA3, and hilus of mice administered with cPTIO (1 mg/kg, i.p., 1 month) or vehicle. Student's *t*-test, n = 4-8 mice. (**C**) Data graphs showing brain-wide excitatory inputs onto DGCs after long-term clearance of NO. Student's *t*-test, n = 4-8 mice. (**D**) Data graphs showing the levels of NO in the DG after long-term clearance of NO. Student's *t*-test, n = 4-6 mice. Error bars correspond to ± s.e.m. *$P < 0.05$, **$P < 0.01$, ****$P < 0.0001$, *NS*, not significant.

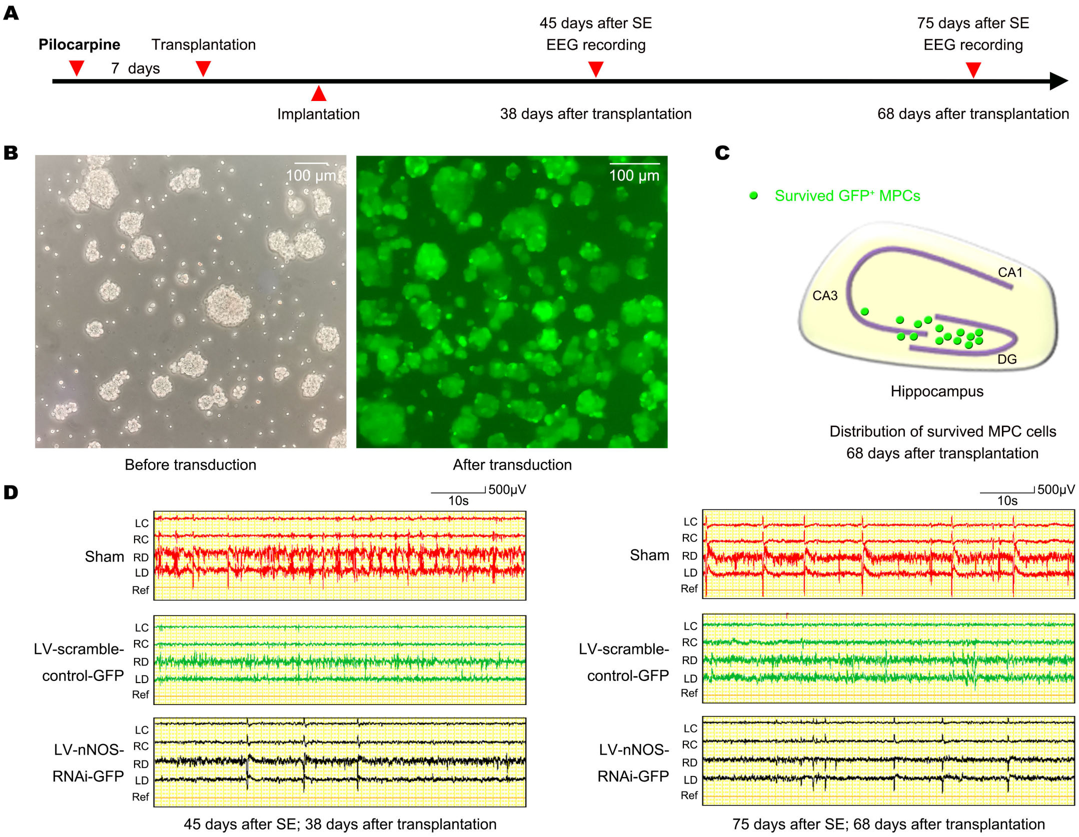

Appendix Figure S3. Transplantation of MPCs lacking nNOS into the hilus. (**A**) Timeline showing the experimental design. (**B**) Representative cultured MPCs infected with LV-nNOS-RNAi-GFP. (**C**) An illustration showing the distribution of surviving GFP$^+$ MPCs 68 days after transplantation. (**D**) Representative EEG record 38 days and 68 days after transplantation of MGE cells infected with LV-scramble-control-GFP or LV-nNOS-RNAi-GFP into the bilateral hilus of mice 7 days post SE induction. LC: left cortex; RC: right cortex; RD: right DG; LD: left DG; Ref: Reference.

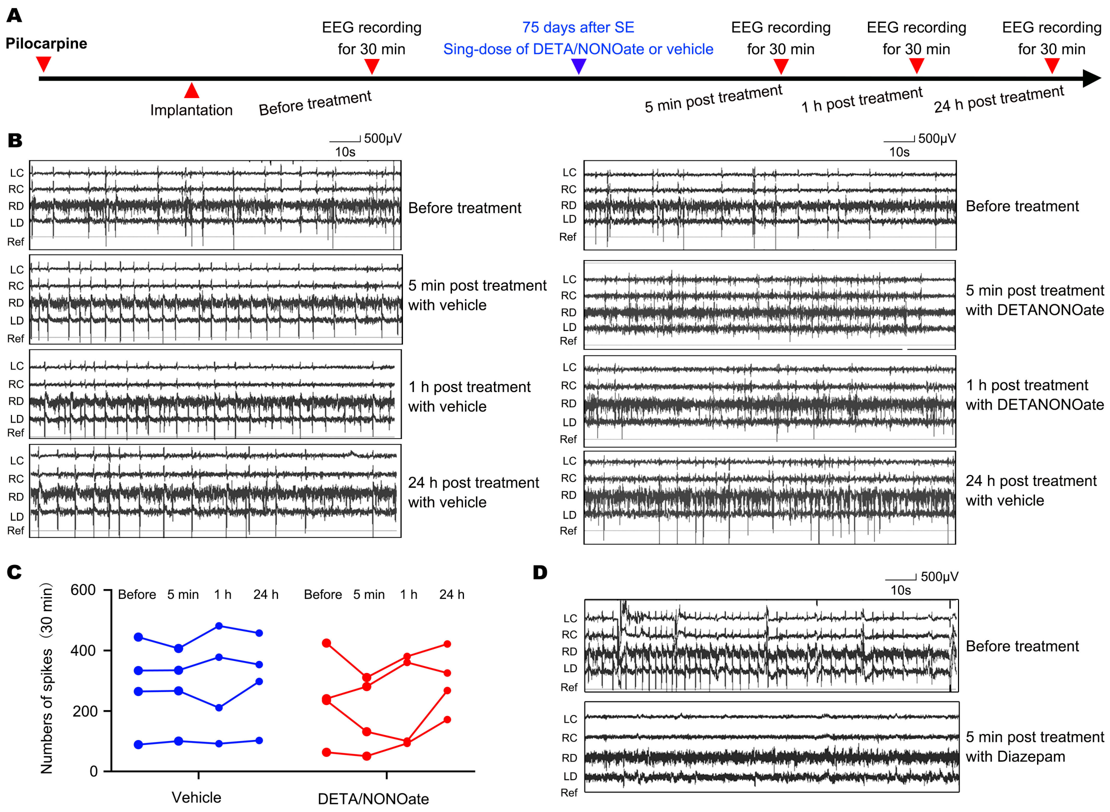

Appendix Figure S4. No significant acute antiseizure effect of DETA/NONOate.

(**A-C**) Experimental paradigm (**A**), representative EEG recording (**B**), and data graph (**C**), showing acute treatment with DETA/NONOate (1 mg/kg, i.p., single-dose) 2.5 months post pilocarpine-induced SE. The EEG was recorded for 30 minutes at 5 minutes, 1 hour, or 24 hours after DETA/NONOate or vehicle administration. LC: left cortex; RC: right cortex; RD: right DG; LD: left DG; Ref: Reference. Two-way ANOVA, n=4. (**D**) Representative EEG recording showing immediate anticonvulsive seizure effect of diazepam (1 mg/kg, i.p., single-dose). LC: left cortex; RC: right cortex; RD: right DG; LD: left DG; Ref: Reference.

Statistics tables

Appendix Table S1

		Test	Group	Method	N	t/F	df	p/q
Figure 1	1D	RNA-Seq	Control	Unpaired Student's *t*-test	n = 6 biologically independent samples	4.221	10	0.0018
			DRE		n = 6 biologically independent samples			
	1E	RT-qPCR	Control	Unpaired Student's *t*-test	n = 6 biologically independent samples	5.083	10	0.0005
			DRE		n = 6 biologically independent samples			
	1F	WB	Control	Unpaired Student's *t*-test	n = 6 biologically independent samples	12.67	10	< 0.0001
			DRE		n = 6 biologically independent samples			
	1G	3h	Control	Two-Way ANOVA with Sidak's multiple comparisons test	n = 5 biologically independent samples	Interaction F(7,61)=14.25 P < 0.0001		0.9996
			Seizure		n = 5 biologically independent samples			
		12h	Control		n = 4 biologically independent samples			> 0.9999
			Seizure		n = 4 biologically independent samples			
		24h	Control		n = 4 biologically independent samples			> 0.9999
			Seizure		n = 5 biologically independent samples			
		48h	Control		n = 5 biologically independent samples			0.0712
			Seizure		n = 5 biologically independent samples			
		7d	Control		n = 5 biologically independent samples			< 0.0001
			Seizure		n = 5 biologically independent samples			
		14d	Control		n = 5 biologically independent samples			< 0.0001
			Seizure		n = 5 biologically independent samples			
		2m	Control		n = 5 biologically independent samples			< 0.0001
			Seizure		n = 5 biologically independent samples			
		4m	Control		n = 5 biologically independent samples			< 0.0001
			Seizure		n = 5 biologically independent samples			
	1H	iNOS	Control	Unpaired Student's *t*-test	n = 5 biologically independent samples	1.661	8	0.1352
			Seizure		n = 5 biologically independent samples			
		eNOS	Control	Unpaired Student's *t*-test	n = 5 biologically independent samples	0.4642	8	0.6549
			Seizure		n = 5 biologically independent samples			
	1J	3h	Control	Two-Way ANOVA with Sidak's multiple comparisons test	n = 6 biologically independent samples	Interaction F(7,80)=6.949 P < 0.0001		> 0.9999
			Seizure		n = 6 biologically independent samples			
		12h	Control		n = 6 biologically independent samples			0.7964
			Seizure		n = 6 biologically independent samples			
		24h	Control		n = 6 biologically independent samples			0.9987
			Seizure		n = 6 biologically independent samples			
		48h	Control		n = 6 biologically independent samples			0.7398
			Seizure		n = 6 biologically independent samples			
		7d	Control		n = 6 biologically independent samples			0.0012
			Seizure		n = 6 biologically independent samples			

		14d	Control		n = 6 biologically independent samples			< 0.0001
			Seizure		n = 6 biologically independent samples			
		2m	Control		n = 6 biologically independent samples			< 0.0001
			Seizure		n = 6 biologically independent samples			
		4m	Control		n = 6 biologically independent samples			< 0.0001
			Seizure		n = 6 biologically independent samples			
	**1K**	Hilus	Control	Unpaired Student's *t*-test	n = 8 biologically independent samples	5.886	14	< 0.0001
			Seizure		n = 8 biologically independent samples			
		CA1	Control	Unpaired Student's *t*-test	n = 8 biologically independent samples	1.134	14	0.2033
			Seizure		n = 8 biologically independent samples			
		CA3	Control	Unpaired Student's *t*-test	n = 8 biologically independent samples	1.516	14	0.1517
			Seizure		n = 8 biologically independent samples			

Appendix Table S2

		Test	Group	Method	N	t/F	df	p/q
**Figure 2**	**2A**	Cumulative seizure score	WT	Unpaired, nonparametric, Kolmogorov-Smirnov test	n = 10 biologically independent samples	N/A	N/A	< 0.0001
			Nos1$^{-/-}$		n = 10 biologically independent samples			
	**2B**	Cumulative seizure score	WT	Unpaired, nonparametric, Kolmogorov-Smirnov test	n = 10 biologically independent samples	N/A	N/A	< 0.0001
			Nos1$^{-/-}$		n = 10 biologically independent samples			
	**2C**	Spikes	WT	Unpaired, Student's *t*-test	n = 6 biologically independent samples	13.05	10	< 0.0001
			Nos1$^{-/-}$		n = 6 biologically independent samples			
	**2D**	Spikes	WT	Unpaired, Student's *t*-test	n = 5 biologically independent samples	3.788	8	0.0053
			nNos$^{-/-}$		n = 5 biologically independent samples			
		SRS	WT	Unpaired, Student's *t*-test	n = 5 biologically independent samples	4.811	8	0.0013
			Nos1$^{-/-}$		n = 5 biologically independent samples			

Appendix Table S3

		Test	Group	Method	N	t/F	df	p/q
Figure 3	3G	Cumulative seizure score	WT	Unpaired, nonparametric, Kolmogorov-Smirnov test	n = 5 biologically independent samples	N/A	N/A	0.8095
			Nos1$^{loxp/loxp}$		n = 5 biologically independent samples			
	3H	Cumulative seizure score	WT	Unpaired, nonparametric, Kolmogorov-Smirnov test	n = 4 biologically independent samples	N/A	N/A	0.0286
			Nos1$^{loxp/loxp}$		n = 4 biologically independent samples			
	3I	Spikes	WT	Unpaired Student's *t*-test	n = 5 biologically independent samples	1.445	8	0.1864
			Nos1$^{loxp/loxp}$		n = 5 biologically independent samples			
		SRS	WT	Nonparametric unpaired Student's *t*-test	n = 5 biologically independent samples	N/A	N/A	N/A
			Nos1$^{loxp/loxp}$		n = 5 biologically independent samples			
	3J	Spikes	WT	Unpaired Student's *t*-test	n = 5 biologically independent samples	5.318	8	0.0007
			Nos1$^{loxp/loxp}$		n = 5 biologically independent samples			
		SRS	WT	Nonparametric unpaired Student's *t*-test	n = 5 biologically independent samples	2.828	8	0.0222
			Nos1$^{loxp/loxp}$		n = 5 biologically independent samples			

Appendix Table S4

		Test	Group	Method	N	t/F	df	p/q
Figure 4	4C	cortex	WT	Unpaired Student's *t*-test	n = 6 biologically independent samples	4.729	10	0.0008
			Nos1$^{-/-}$		n = 6 biologically independent samples			
		forebrain	WT	Unpaired Student's *t*-test	n = 6 biologically independent samples	5.461	10	0.0003
			Nos1$^{-/-}$		n = 6 biologically independent samples			
		brainstem	WT	Unpaired Student's *t*-test	n = 6 biologically independent samples	3.096	10	0.0113
			Nos1$^{-/-}$		n = 6 biologically independent samples			
		CA3	WT	Unpaired Student's *t*-test	n = 6 biologically independent samples	5.290	10	0.0004
			Nos1$^{-/-}$		n = 6 biologically independent samples			
		HL	WT	Unpaired Student's *t*-test	n = 6 biologically independent samples	1.308	10	0.2201
			Nos1$^{-/-}$		n = 6 biologically independent samples			
	4F	cortex	WT	Unpaired Student's *t*-test	n = 7 biologically independent samples	15.67	11	< 0.0001
			Nos1$^{loxp/loxp}$		n = 6 biologically independent samples			
		forebrain	WT	Unpaired Student's *t*-test	n = 7 biologically independent samples	8.713	11	< 0.0001
			Nos1$^{loxp/loxp}$		n = 6 biologically independent samples			
		brainstem	WT	Unpaired Student's *t*-test	n = 7 biologically independent samples	6.005	11	< 0.0001
			Nos1$^{loxp/loxp}$		n = 6 biologically independent samples			
		CA3	WT	Unpaired Student's *t*-test	n = 7 biologically independent samples	11.64	11	< 0.0001
			Nos1$^{loxp/loxp}$		n = 6 biologically independent samples			
		HL	WT	Unpaired Student's *t*-test	n = 7 biologically independent samples	0.1651	11	0.8718
			Nos1$^{loxp/loxp}$		n = 6 biologically independent samples			
	3G	Excitatory Input cells	WT	Unpaired Student's *t*-test	n = 7 biologically independent samples	16.57	11	< 0.0001
			Nos1$^{loxp/loxp}$		n = 6 biologically independent samples			

Appendix Table S5

		Test	Group	Method	N	t/F	df	p/q
Figure 5	5A	GL	WT	Unpaired Student's *t*-test	n = 8 biologically independent samples	3.688	14	0.0024
			Nos1$^{-/-}$		n = 8 biologically independent samples			
		HL	WT	Unpaired Student's *t*-test	n = 8 biologically independent samples	1.036	14	0.3176
			Nos1$^{-/-}$		n = 8 biologically independent samples			
	5B	CA3	WT	Unpaired Student's *t*-test	n = 8 biologically independent samples	0.4303	14	0.6735
			Nos1$^{-/-}$		n = 8 biologically independent samples			
		CA1	WT	Unpaired Student's *t*-test	n = 8 biologically independent samples	0.7037	14	0.4931
			Nos1$^{-/-}$		n = 8 biologically independent samples			
	5C	mEPSC frequency	WT	Unpaired Student's *t*-test	n = 12 cells from 3 mice	2.583	22	0.0170
			Nos1$^{-/-}$		n = 12 cells from 3 mice			
		mEPSC amplitude	WT	Unpaired Student's *t*-test	n = 12 cells from 3 mice	1.658	22	0.1114
			Nos1$^{-/-}$		n = 12 cells from 3 mice			
	5D	mIPSC frequency	WT	Unpaired Student's *t*-test	n = 15 cells from 3 mice	2.040	28	0.0509
			Nos1$^{-/-}$		n = 15 cells from 3 mice			
		mIPSC amplitude	WT	Unpaired Student's *t*-test	n = 15 cells from 3 mice	1.176	28	0.2494
			Nos1$^{-/-}$		n = 15 cells from 3 mice			
	5E	50 ms	WT	Two-way ANOVA	n = 4 cells from 3 mice	Genetic factor $F(1,32)=10.47$ $P=0.0028$		0.0419
			Nos1$^{-/-}$		n = 6 cells from 3 mice			
		100 ms	WT		n = 4 cells from 3 mice			0.5420
			Nos1$^{-/-}$		n = 6 cells from 3 mice			
		200 ms	WT		n = 4 cells from 3 mice			0.9994
			Nos1$^{-/-}$		n = 6 cells from 3 mice			
		300 ms	WT		n = 4 cells from 3 mice			0.1383
			Nos1$^{-/-}$		n = 6 cells from 3 mice			
	5F	50 ms	WT	Two-way ANOVA	n = 7 cells from 3 mice	Genetic factor $F(1,40)= 0.2523$ $P=0.6182$		0.8910
			Nos1$^{-/-}$		n = 6 cells from 3 mice			
		100 ms	WT		n = 7 cells from 3 mice			0.8898
			Nos1$^{-/-}$		n = 6 cells from 3 mice			
		200 ms	WT		n = 7 cells from 3 mice			0.9994
			Nos1$^{-/-}$		n = 6 cells from 3 mice			
		300 ms	WT		n = 7 cells from 3 mice			0.9897
			Nos1$^{-/-}$		n = 6 cells from 3 mice			
	5G	Input resistance	WT	Unpaired Student's *t*-test	n = 18 cells from 3 mice	0.02615	34	0.9793
			Nos1$^{-/-}$		n = 18 cells from 3 mice			
		Membrane Potential	WT	Unpaired Student's *t*-test	n = 18 cells from 3 mice	0.2590	34	0.7972
			Nos1$^{-/-}$		n = 18 cells from 3 mice			
		Minimal Current	WT	Unpaired Student's *t*-test	n = 18 cells from 3 mice	0.3378	34	0.7376
			Nos1$^{-/-}$		n = 18 cells from 3 mice			
		Spike 0	WT	Two-way ANOVA	n = 18 cells from 3 mice	Interaction		> 0.9999

		Number		*Nos1*$^{-/-}$	with Sidak's multiple comparisons test	n = 18 cells from 3 mice	F(10,374)= 0.08814 P=0.9999	
			10	WT		n = 18 cells from 3 mice		> 0.9999
				Nos1$^{-/-}$		n = 18 cells from 3 mice		
			20	WT		n = 18 cells from 3 mice		> 0.9999
				Nos1$^{-/-}$		n = 18 cells from 3 mice		
			30	WT		n = 18 cells from 3 mice		> 0.9999
				Nos1$^{-/-}$		n = 18 cells from 3 mice		
			40	WT		n = 18 cells from 3 mice		> 0.9999
				Nos1$^{-/-}$		n = 18 cells from 3 mice		
			50	WT		n = 18 cells from 3 mice		> 0.9999
				Nos1$^{-/-}$		n = 18 cells from 3 mice		
			60	WT		n = 18 cells from 3 mice		> 0.9999
				Nos1$^{-/-}$		n = 18 cells from 3 mice		
			70	WT		n = 18 cells from 3 mice		> 0.9999
				Nos1$^{-/-}$		n = 18 cells from 3 mice		
			80	WT		n = 18 cells from 3 mice		> 0.9999
				Nos1$^{-/-}$		n = 18 cells from 3 mice		
			90	WT		n = 18 cells from 3 mice		> 0.9999
				Nos1$^{-/-}$		n = 18 cells from 3 mice		
			100	WT		n = 18 cells from 3 mice		> 0.9999
				Nos1$^{-/-}$		n = 18 cells from 3 mice		

Appendix Table S6

		Test	Group	Method	N	t/F	df	p/q
Figure 6	6A	GFP^{+}	LV-CON-GFP	Unpaired, Student's *t*-test	n = 6 biologically independent samples	0.1515	10	0.8826
			LV-nNOS-GFP		n = 6 biologically independent samples			
	6B	HL	LV-CON-GFP	Unpaired, Student's *t*-test	n = 6 biologically independent samples	4.749	10	0.0008
			LV-nNOS-GFP		n = 6 biologically independent samples			
		GL+ML	LV-CON-GFP	Unpaired, Student's *t*-test	n = 6 biologically independent samples	1.632	10	0.1338
			LV-nNOS-GFP		n = 6 biologically independent samples			
	6C	nNOS/ GAPDH	Control vs. Seizure (LV-CON-GFP)	One-Way ANOVA	n = 8 biologically independent samples	Interaction F(2,21)=24.38 P < 0.0001	< 0.0001	
			Seizure (LV-CON-GFP) vs Seizure (LV-nNOS-GFP)		n = 8 biologically independent samples		0.9937	
			Control vs. Seizure (LV-nNOS-GFP)		n = 8 biologically independent samples		< 0.0001	
		NO level	Control vs. Seizure (LV-CON-GFP)	One-Way ANOVA	n = 7 biologically independent samples	Interaction F(2,17)=69.29 P < 0.0001	< 0.0001	
			Seizure (LV-CON-GFP) vs Seizure (LV-nNOS-GFP)		n = 7 biologically independent samples		< 0.0001	
			Control vs. Seizure (LV-nNOS-GFP)		n = 6 biologically independent samples		< 0.0001	
	6D	Spikes	Control vs. Seizure (LV-CON-GFP)	One-Way ANOVA	n = 6 biologically independent samples	Interaction F(2,15)=55.02 P < 0.0001	< 0.0001	
			Seizure (LV-CON-GFP) vs Seizure (LV-nNOS-GFP)		n = 6 biologically independent samples		< 0.0001	
			Control vs. Seizure (LV-nNOS-GFP)		n = 6 biologically independent samples		0.0707	
		SRS	Control vs. Seizure (LV-CON-GFP)	Kruskal-Wallis test	n = 6 biologically independent samples	Corrected method of Benjamini and yekutieli	0.0003	
			Seizure (LV-CON-GFP) vs. Seizure (LV-nNOS-GFP)		n = 6 biologically independent samples		0.0092	
			Control vs. Seizure (LV-nNOS-GFP)		n = 6 biologically independent samples		0.2976	
	6F	Latency (Day 5)	Control+LV-GFP	One-Way ANOVA	n = 22 biologically independent samples	F(3,65)=7.457 P=0.0002	Seziure+LV-GFP Vs. Seziure+LV-nNOS-GFP P=0.0146	
			Seziure+LV-GFP		n = 15 biologically independent samples			
			Seziure+LV-nNOS-GFP		n = 13 biologically independent samples			
			Seziure+LV-nNOSΔ-GFP		n = 19 biologically independent samples			
	6G	Target	Control+LV-GFP	One-Way ANOVA	n = 23 biologically independent samples	F(3,68)=7.179 P=0.0003	Control+LV-GFP Vs. Seziure+LV-GFP P=0.0106	
			Seziure+LV-GFP		n = 15 biologically independent samples		Seziure+LV-GFP Vs. Seziure+LV-	

							nNOS-GFP P=0.0411
			Seziure+LV-nNOS-GFP		n = 15 biologically independent samples		Seziure+LV-GFP Vs. Seziure+LV-nNOSΔ-GFP P=0.9915
			Seziure+LV-nNOSΔ-GFP		n = 19 biologically independent samples		Seziure+LV-nNOS-GFP Vs. Seziure+LV-nNOSΔ-GFP P=0.0124
		Speed	Control+LV-GFP	One-Way ANOVA	n = 23 biologically independent samples	F(3,68)=3.233 P=0.0276	Control+LV-GFP Vs. Seziure+LV-GFP P=0.3647
			Seziure+LV-GFP		n = 15 biologically independent samples		Seziure+LV-GFP Vs. Seziure+LV-nNOS-GFP P=0.0651
			Seziure+LV-nNOS-GFP		n = 15 biologically independent samples		Seziure+LV-GFP Vs. Seziure+LV-nNOSΔ-GFP P > 0.9999
			Seziure+LV-nNOSΔ-GFP		n = 19 biologically independent samples		Seziure+LV-nNOS-GFP Vs. Seziure+LV-nNOSΔ-GFP P=0.0512
	**6J**	Spontaneous alteration	Control+LV-GFP	One-Way ANOVA	n = 15 biologically independent samples	F(3,56)=8.807 P < 0.0001	Control+LV-GFP Vs. Seziure+LV-GFP P=0.0090
			Seziure+LV-GFP		n = 15 biologically independent samples		Seziure+LV-GFP Vs. Seziure+LV-nNOS-GFP P=0.0331
			Seziure+LV-nNOS-GFP		n = 15 biologically independent samples		Seziure+LV-GFP Vs. Seziure+LV-nNOSΔ-GFP P=0.7558
			Seziure+LV-nNOSΔ-GFP		n = 15 biologically independent samples		Seziure+LV-nNOS-GFP Vs. Seziure+LV-nNOSΔ-GFP

	6L						P=0.0020
		Time in new arm	Control+LV-GFP	One-Way ANOVA	n = 15 biologically independent samples	F(3,56)=10.03 P < 0.0001	Control+LV-GFP Vs. Seziure+LV-GFP P=0.0087
			Seziure+LV-GFP		n = 15 biologically independent samples		Seziure+LV-GFP Vs. Seziure+LV-nNOS-GFP P=0.0015
			Seziure+LV-nNOS-GFP		n = 15 biologically independent samples		Seziure+LV-GFP Vs. Seziure+LV-nNOSΔ-GFP P=0.9606
			Seziure+LV-nNOSΔ-GFP		n = 15 biologically independent samples		Seziure+LV-nNOS-GFP Vs. Seziure+LV-nNOSΔ-GFP P=0.0003
	**6L**	ORM	Control+LV-GFP	One-Way ANOVA	n = 15 biologically independent samples	F(3,56)=7.320 P=0.0003	Control+LV-GFP Vs. Seziure+LV-GFP P=0.0281
			Seziure+LV-GFP		n = 15 biologically independent samples		Seziure+LV-GFP Vs. Seziure+LV-nNOS-GFP P=0.0338
			Seziure+LV-nNOS-GFP		n = 15 biologically independent samples		Seziure+LV-GFP Vs. Seziure+LV-nNOSΔ-GFP P=0.8373
			Seziure+LV-nNOSΔ-GFP		n = 15 biologically independent samples		Seziure+LV-nNOS-GFP Vs. Seziure+LV-nNOSΔ-GFP P=0.0032
		OLM	Control+LV-GFP	One-Way ANOVA	n = 15 biologically independent samples	F(3,56)=8.608 P < 0.0001	Control+LV-GFP Vs. Seziure+LV-GFP P=0.0118
			Seziure+LV-GFP		n = 15 biologically independent samples		Seziure+LV-GFP Vs. Seziure+LV-nNOS-GFP P=0.0247

			Seziure+LV-nNOS-GFP		n = 15 biologically independent samples		Seziure+LV-GFP Vs. Seziure+LV-nNOSΔ-GFP P=0.7904
			Seziure+LV-nNOSΔ-GFP		n = 15 biologically independent samples		Seziure+LV-nNOS-GFP Vs. Seziure+LV-nNOSΔ-GFP P=0.0017
	**6N**	45 days	Sham vs. LV-scramble-control-GFP	Brown-Forsythe and Welch's ANOVA tests	n = 3 biologically independent samples	F(2, 6.150)= 32.34 P=0.0005	0.0179
			LV-scramble-control-GFP vs. LV-nNOS-RNAi-GFP		n = 4 biologically independent samples		0.0200
			Sham vs. LV-nNOS-RNAi-GFP		n = 5 biologically independent samples		0.0382
		75 days	Sham vs. LV-scramble-control-GFP	Brown-Forsythe and Welch's ANOVA tests	n = 3 biologically independent samples	F(2, 2.388)= 58.81 P=0.0005	0.0286
			LV-scramble-control-GFP vs. LV-nNOS-RNAi-GFP		n = 3 biologically independent samples		0.0370
			Sham vs. LV-nNOS-RNAi-GFP		n = 5 biologically independent samples		0.0423

Appendix Table S7

		Test	Group	Method	N	t/F	df	p/q
Figure 7	7C	Cortex	Control + Vehicle vs SE + Vehicle	Brown-Forsythe and Welch ANOVA tests	n = 6 biologically independent samples	F(2, 10.73)=23.77 P=0.0001		0.0049
			SE + Vehicle vs SE + DETA/NONOate		n = 6 biologically independent samples			0.0005
			Control + Vehicle SE + DETA/NONOate		n = 6 biologically independent samples			0.5917
		Forebrain	Control + Vehicle vs SE + Vehicle	Brown-Forsythe and Welch ANOVA tests	n = 6 biologically independent samples	F(2, 8.153)=0.5922 P=0.5752		0.4890
			SE + Vehicle vs SE + DETA/NONOate		n = 6 biologically independent samples			0.9949
			Control + Vehicle SE + DETA/NONOate		n = 6 biologically independent samples			0.6446
		Brainstem	Control + Vehicle vs SE + Vehicle	Brown-Forsythe and Welch ANOVA tests	n = 6 biologically independent samples	F(2, 9.051)=0.5935 P=0.5725		0.3562
			SE + Vehicle vs SE + DETA/NONOate		n = 6 biologically independent samples			0.8561
			Control + Vehicle SE + DETA/NONOate		n = 6 biologically independent samples			0.9960
		CA3	Control + Vehicle vs SE + Vehicle	Brown-Forsythe and Welch ANOVA tests	n = 6 biologically independent samples	F(2, 10.45)=2.840 P=0.1035		0.3486
			SE + Vehicle vs SE + DETA/NONOate		n = 6 biologically independent samples			0.1834
			Control + Vehicle SE + DETA/NONOate		n = 6 biologically independent samples			0.6206
		Hilus	Control + Vehicle vs SE + Vehicle	Brown-Forsythe and Welch ANOVA tests	n = 6 biologically independent samples	F(2, 14.52)=7.510 P=0.0058		0.0057
			SE + Vehicle vs SE + DETA/NONOate		n = 6 biologically independent samples			0.2434
			Control + Vehicle SE + DETA/NONOate		n = 6 biologically independent samples			0.2203
		Excitatory input cells	Control + Vehicle vs SE + Vehicle	Brown-Forsythe and Welch ANOVA tests	n = 6 biologically independent samples	F(2, 9.188)=33.80 P < 0.0001		0.0004
			SE + Vehicle vs SE + DETA/NONOate		n = 6 biologically independent samples			0.0007
			Control + Vehicle SE + DETA/NONOate		n = 6 biologically independent samples			0.2915
	7F	Spikes	Vehicle (Control)	Two-Way ANOVA	n = 6 biologically independent samples	Drug factor F(3,15)=47.82 P < 0.0001		DETA/NONOate: Control Vs. Pilocarpine P > 0.9999
			DETA/NONOate (Control)		n = 6 biologically independent samples			

			Vehicle (Pilocarpine)		n = 6 biologically independent samples		Pilocarpine: Vehicle Vs. DETA/NONOate $P < 0.0001$
			DETA/NONOate (Pilocarpine)		n = 6 biologically independent samples		
		SRS	Vehicle (Control)	Two-Way ANOVA	n = 6 biologically independent samples	Drug factor $F(3,15)=36.50$ $P < 0.0001$	DETA/NONOate: Control Vs. Pilocarpine $P > 0.9999$
			DETA/NONOate (Control)		n = 6 biologically independent samples		
			Vehicle (Pilocarpine)		n = 6 biologically independent samples		Pilocarpine: Vehicle Vs. DETA/NONOate $P < 0.0001$
			DETA/NONOate (Pilocarpine)		n = 6 biologically independent samples		
	**7G**	NO level	Control+Vehicle Vs SE+Vehicle	One-Way ANOVA	n = 6 biologically independent samples	$F(2,15)=55.22$ $P < 0.0001$	< 0.0001
			SE+Vehicle Vs SE+DETA/NONOate		n = 6 biologically independent samples		0.0495
			Control+Vehicle Vs SE+DETA/NONOate		n = 6 biologically independent samples		< 0.0001

Appendix Table S8

		Test	Group	Method	N	t/F	df	p/q
Expanded View Figure 1	EV 1A	7d	Control	Unpaired, Student's *t*-test	n = 4 biologically independent samples	2.455	6	0.0247
			Seizure		n = 4 biologically independent samples			
		2m	Control	Unpaired, Student's *t*-test	n = 5 biologically independent samples	4.849	8	0.0013
			Seizure		n = 5 biologically independent samples			
	EV 1B	NO level	Control vs SE 7d	One-Way ANOVA	n = 4 biologically independent samples	F(2, 9)=23.34 P=0.0003		0.0016
			Control vs SE 60d		n = 4 biologically independent samples			0.0003
			SE 7d vs SE 60d		n = 4 biologically independent samples			0.4040
	EV 1E	nNOS/Actin	Vehicle	Unpaired, Student's *t*-test	n = 7 biologically independent samples	4.472	12	0.0008
			KA		n = 7 biologically independent samples			
		NO level	Vehicle	Unpaired, Student's *t*-test	n = 9 biologically independent samples	5.736	16	$<$ 0.0001
			KA		n = 9 biologically independent samples			
	EV 1F	nNOS/Actin	Vehicle	Unpaired, Student's *t*-test	n = 6 biologically independent samples	2.648	8	0.0293
			PTZ		n = 4 biologically independent samples			
		NO level	Vehicle	Unpaired, Student's *t*-test	n = 6 biologically independent samples	2.160	14	0.0486
			PTZ		n = 10 biologically independent samples			

Appendix Table S9

<table>
<tr><th></th><th></th><th>Test</th><th>Group</th><th>Method</th><th>N</th><th>t/F</th><th>df</th><th>p/q</th></tr>
<tr><td rowspan="12">Expanded View Figure 2</td><td rowspan="4">EV 2A</td><td rowspan="2">GFP</td><td>Scramble</td><td rowspan="2">Unpaired, Student's t-test</td><td>n = 8 biologically independent samples</td><td rowspan="2">0.4157</td><td rowspan="2">14</td><td rowspan="2">0.6839</td></tr>
<tr><td>RNAi</td><td>n = 8 biologically independent samples</td></tr>
<tr><td rowspan="2">nNOS</td><td>Scramble</td><td rowspan="2">Unpaired, Student's t-test</td><td>n = 8 biologically independent samples</td><td rowspan="2">6.3000</td><td rowspan="2">14</td><td rowspan="2">< 0.0001</td></tr>
<tr><td>RNAi</td><td>n = 8 biologically independent samples</td></tr>
<tr><td rowspan="4">EV 2B</td><td rowspan="2">Total DG</td><td>Scramble</td><td rowspan="2">Unpaired, Student's t-test</td><td>n = 8 biologically independent samples</td><td rowspan="2">5.642</td><td rowspan="2">14</td><td rowspan="2">< 0.0001</td></tr>
<tr><td>RNAi</td><td>n = 8 biologically independent samples</td></tr>
<tr><td rowspan="2">ML+GL</td><td>Scramble</td><td rowspan="2">Unpaired, Student's t-test</td><td>n = 8 biologically independent samples</td><td rowspan="2">4.071</td><td rowspan="2">14</td><td rowspan="2">0.0011</td></tr>
<tr><td>RNAi</td><td>n = 8 biologically independent samples</td></tr>
<tr><td rowspan="2">EV 2C</td><td rowspan="2">Cumulative seizure score</td><td>Scramble</td><td rowspan="2">Unpaired, nonparametric, Kolmogorov-Smirnov test</td><td>n = 8 biologically independent samples</td><td rowspan="2">N/A</td><td rowspan="2">N/A</td><td rowspan="2">0.0186</td></tr>
<tr><td>RNAi</td><td>n = 8 biologically independent samples</td></tr>
<tr><td rowspan="4">EV 2D</td><td rowspan="2">Spikes</td><td>Scramble</td><td rowspan="2">Unpaired, Student's t-test</td><td>n = 3 biologically independent samples</td><td rowspan="2">6.133</td><td rowspan="2">4</td><td rowspan="2">0.0036</td></tr>
<tr><td>RNAi</td><td>n = 3 biologically independent samples</td></tr>
<tr><td rowspan="2">SRS</td><td>Scramble</td><td rowspan="2">Unpaired, Student's t-test</td><td>n = 3 biologically independent samples</td><td rowspan="2">7.071</td><td rowspan="2">4</td><td rowspan="2">0.0021</td></tr>
<tr><td>RNAi</td><td>n = 3 biologically independent samples</td></tr>
</table>

Appendix Table S10

		Test	Group	Method	N	t/F	df	p/q
Expanded View Figure 3	EV 3C	GFP⁺ cells	WT	Unpaired, Student's *t*-test	n = 3 biologically independent samples	0.0874	4	0.9346
			Nos1$^{loxp/loxp}$		n = 3 biologically independent samples			
	EV 3D	nNOS⁺ cells in the hilus	WT	Unpaired, Student's *t*-test	n = 3 biologically independent samples	0..3825	4	0.7215
			Nos1$^{loxp/loxp}$		n = 3 biologically independent samples			
	EV 3E	nNOS/GAPDH	WT	Unpaired, Student's *t*-test	n = 6 biologically independent samples	13.83	10	$<$ 0.0001
			Nos1$^{loxp/loxp}$		n = 6 biologically independent samples			
	EV 3F	nNOS⁺&GFP⁺ /GFP⁺	WT	Unpaired, Student's *t*-test	n = 3 biologically independent samples	18.56	4	$<$ 0.0001
			Nos1$^{loxp/loxp}$		n = 3 biologically independent samples			
	EV 3G	nNOS/GAPDH	WT	Unpaired, Student's *t*-test	n = 6 biologically independent samples	8.509	10	$<$ 0.0001
			Nos1$^{loxp/loxp}$		n = 6 biologically independent samples			

Appendix Table S11

		Test	Group	Method	N	t/F	df	p/q
**Expanded View Figure 5**	**EV 5A**	ALT	Vehicle (Control)	Two-Way ANOVA	n = 6 biologically independent samples	Drug factor F(3,15)=1.294 P=0.3127		DETA/NONOate: Control Vs. Pilocarpine P=0.9981
			DETA/NONOate (Control)		n = 6 biologically independent samples			
			Vehicle - (Pilocarpine)		n = 6 biologically independent samples			Control: Vehicle Vs. DETA/NONOate P=0.3823
			DETA/NONOate (Pilocarpine)		n = 6 biologically independent samples			Pilocarpine: Vehicle Vs. DETA/NONOate P=0.9972
		Cr	Vehicle (Control)	Two-Way ANOVA	n = 6 biologically independent samples	Drug factor F(3,15)=0.05222 P=0.9836		DETA/NONOate: Control Vs. Pilocarpine P=0.9998
			DETA/NONOate (Control)		n = 6 biologically independent samples			Control: Vehicle Vs. DETA/NONOate P=0.9926
			Vehicle (Pilocarpine)		n = 6 biologically independent samples			Pilocarpine: Vehicle Vs. DETA/NONOate P=0.9938
			DETA/NONOate (Pilocarpine)		n = 6 biologically independent samples			
		CTn	Vehicle (Control)	Two-Way ANOVA	n = 6 biologically independent samples	Drug factor F(3,15)=0.6148 P=0.6159		DETA/NONOate: Control Vs. Pilocarpine P=0.5492
			DETA/NONOate (Control)		n = 6 biologically independent samples			
			Vehicle (Pilocarpine)		n = 6 biologically independent samples			Control: Vehicle Vs. DETA/NONOate P=0.9520
			DETA/NONOate (Pilocarpine)		n = 6 biologically independent samples			Pilocarpine: Vehicle Vs. DETA/NONOate P=0.8821
		TBIL	Vehicle (Control)	Two-Way ANOVA	n = 6 biologically independent samples	Drug factor F(3,15)=2.601 P=0.0905		DETA/NONOate: Control Vs. Pilocarpine P=0.9287
			DETA/NONOate (Control)		n = 6 biologically independent samples			Control: Vehicle Vs. DETA/NONOate P=0.3798
			Vehicle (Pilocarpine)		n = 6 biologically independent samples			Pilocarpine: Vehicle Vs. DETA/NONOate

				DETA/NONOate (Pilocarpine)		n = 6 biologically independent samples		P=0.1680
		LDH		Vehicle (Control)	Two-Way ANOVA	n = 6 biologically independent samples	Drug factor F(3,15)=1.580 P=0.2357	DETA/NONOate: Control Vs. Pilocarpine P=0.5614
				DETA/NONOate (Control)		n = 6 biologically independent samples		Control: Vehicle Vs. DETA/NONOate P=0.9689
				Vehicle (Pilocarpine)		n = 6 biologically independent samples		Pilocarpine: Vehicle Vs. DETA/NONOate P=0.9997
				DETA/NONOate (Pilocarpine)		n = 6 biologically independent samples		
	**EV 5D**	Spikes	30d	Vehicle	Two-Way ANOVA	n = 6 biologically independent samples	Interaction F(2,30)=4.625 P=0.0178	45d: Vehicle Vs. DETA/NONOate P=0.0058
				DETA/NONOate		n = 6 biologically independent samples		75d: Vehicle Vs. DETA/NONOate P=0.0425
			45d	Vehicle		n = 6 biologically independent samples		DETA/NONOate: 30d Vs. 45d P=0.0022
				DETA/NONOate		n = 6 biologically independent samples		
			75d	Vehicle		n = 6 biologically independent samples		DETA/NONOate: 30d Vs. 75d P < 0.0001
				DETA/NONOate		n = 6 biologically independent samples		
		SRS	30d	Vehicle	Two-Way ANOVA	n = 6 biologically independent samples	Interaction F(2,30)=4.766 P=0.0159	75d: Vehicle Vs. DETA/NONOate P=0.0034
				DETA/NONOate		n = 6 biologically independent samples		
			45d	Vehicle		n = 6 biologically independent samples		
				DETA/NONOate		n = 6 biologically independent samples		Vehicle: 30d Vs.75d P < 0.0001
			75d	Vehicle		n = 6 biologically independent samples		
				DETA/NONOate		n = 6 biologically independent samples		
	**EV 5G**	Spikes	30d	Vehicle	Two-Way ANOVA	n = 6 biologically independent samples	Interaction F(3,40)=10.95 P < 0.0001	45d: Vehicle Vs. DETA/NONOate P < 0.0001
				DETA/NONOate		n = 6 biologically independent samples		75d: Vehicle Vs. DETA/NONOate P < 0.0001
			45d	Vehicle		n = 6 biologically independent samples		90d: Vehicle Vs. DETA/NONOate P < 0.0001
				DETA/NONOate		n = 6 biologically independent samples		
			75d	Vehicle		n = 6 biologically independent samples		DETA/NONOate: 30d Vs. 45d P < 0.0001
				DETA/NONOate		n = 6 biologically independent samples		DETA/NONOate: 30d

								Vs.75d<br>P < 0.0001
			90d	Vehicle		n = 6 biologically independent samples		DETA/NONOate: 30d Vs. 90d<br>P < 0.0001
				DETA/NONOate		n = 6 biologically independent samples		
		SRS	30d	Vehicle	Two-Way ANOVA	n = 6 biologically independent samples	Interaction<br>F(3,40)=8.842<br>P < 0.0001	75d: Vehicle Vs. DETA/NONOate<br>P < 0.0001
				DETA/NONOate		n = 6 biologically independent samples		
			45d	Vehicle		n = 6 biologically independent samples		90d: Vehicle Vs. DETA/NONOate<br>P < 0.0001
				DETA/NONOate		n = 6 biologically independent samples		
			75d	Vehicle		n = 6 biologically independent samples		Vehicle: 30d Vs.75d<br>P < 0.0001
				DETA/NONOate		n = 6 biologically independent samples		
			90d	Vehicle		n = 6 biologically independent samples		Vehicle: 30d Vs.90d<br>P < 0.0001
				DETA/NONOate		n = 6 biologically independent samples		

Appendix Table S12

		Test	Group	Method	N	t/F	df	p/q
**Appendix Figure S1**	**S1C**	Spikes	DG	Unpaired, Student's *t*-test	n = 5 biologically independent samples	10.96	8	< 0.0001
			Cortex		n = 5 biologically independent samples			

Appendix Table S13

		Test	Group	Method	N	t/F	df	p/q
**Appendix Figure S2**	**S2B**	Cortex	Vehicle	Unpaired, Student's *t*-test	n = 4 biologically independent samples	2.332	10	0.0419
			cPTIO		n = 9 biologically independent samples			
		Forebrain	Vehicle	Unpaired, Student's *t*-test	n = 4 biologically independent samples	2.268	10	0.0468
			cPTIO		n = 9 biologically independent samples			
		Brainstem	Vehicle	Unpaired, Student's *t*-test	n = 4 biologically independent samples	3.386	10	0.0069
			cPTIO		n = 9 biologically independent samples			
		CA3	Vehicle	Unpaired, Student's *t*-test	n = 4 biologically independent samples	1.384	10	0.1966
			cPTIO		n = 9 biologically independent samples			
		HL	Vehicle	Unpaired, Student's *t*-test	n = 4 biologically independent samples	2.086	10	0.0635
			cPTIO		n = 9 biologically independent samples			
	**S2C**	Excitatory	Vehicle	Unpaired, Student's *t*-test	n = 4 biologically independent samples	2.692	10	0.0226
			cPTIO		n = 9 biologically independent samples			
	**S2D**	NO	Vehicle	Unpaired, Student's *t*-test	n = 4 biologically independent samples	8.391	8	< 0.0001
			cPTIO		n = 6 biologically independent samples			

Appendix Table S14

		Test		Group	Method	N	t/F	df	p/q
**Appendix Figure S4**	**S4C**	Spikes	Vehicle	Before	Two-Way ANOVA	n = 4 biologically independent samples	Drug factor F(1.953,13.67) =3.270 P=0.07		5 min Vs. Before P=0.5555
				5 min		n = 4 biologically independent samples			
				1 h		n = 4 biologically independent samples			1 h Vs. Before P > 0.9999
				24 h		n = 4 biologically independent samples			
			DETA/NONOate	Before		n = 4 biologically independent samples			24 h Vs. Before P=0.0994
				5 min		n = 4 biologically independent samples			
				1 h		n = 4 biologically independent samples			
				24 h		n = 4 biologically independent samples			
