# Supplementary material for: A novel anti-epileptogenesis strategy of temporal lobe epilepsy based on nitric oxide donor
Source: EMBO Mol Med. 2024 Dec 9;17(1):85–111. doi: 10.1038/s44321-024-00168-1 (PMC11730642; doi:10.1038/s44321-024-00168-1)
Supplement: Supplementary file 2 — Table EV1 [file 44321_2024_168_MOESM2_ESM.docx]

# Expanded View table

**Table EV1. Clinical information of patients (DRE) and body donors (control).**

|  | **Gender** | **Age** | **Disease** | **Treatment with ASDs?** |
| --- | --- | --- | --- | --- |
| **Control 1** | Male | 69 | Cancer (Liver)  Nonepileptic experience | No |
| **Control 2** | Female | 59 | Cancer (Lung)  Nonepileptic experience | No |
| **Control 3** | Male | 51 | Cancer (Liver)  Nonepileptic experience | No |
| **DRE 1** | Male | 39 | DRE | Yes |
| **DRE 2** | Male | 42 | DRE | Yes |
| **DRE 3** | Female | 50 | DRE | Yes |
