# Supplementary figures and images for: A novel anti-epileptogenesis strategy of temporal lobe epilepsy based on nitric oxide donor
Source: EMBO Mol Med. 2024 Dec 9;17(1):85–111. doi: 10.1038/s44321-024-00168-1 (PMC11730642; doi:10.1038/s44321-024-00168-1)

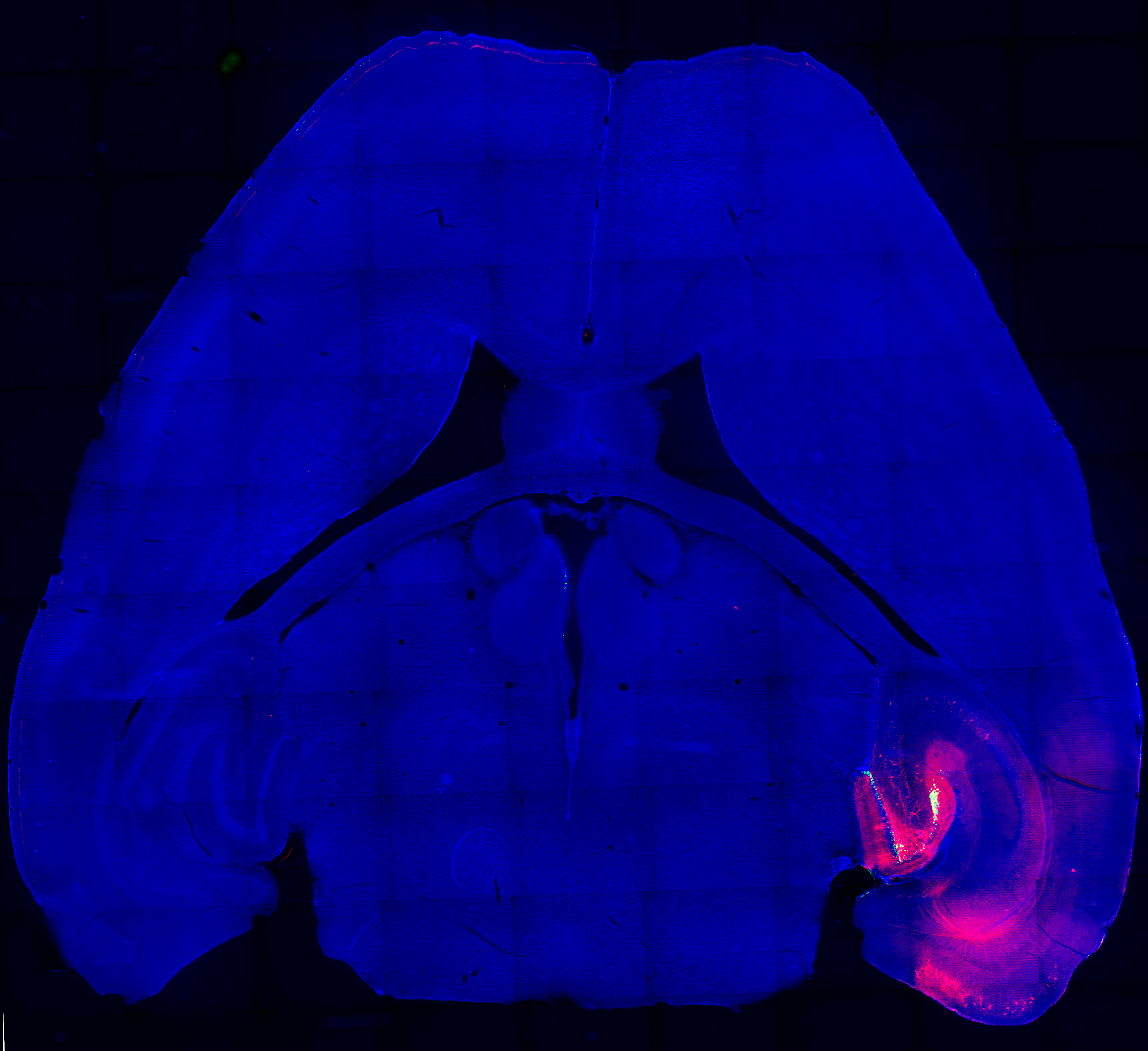

Supplement: Supplementary file 7 — Source data Fig. 4 [file 44321_2024_168_MOESM7_ESM.zip › Figure 4/4A/Nos1-- -1.tif]

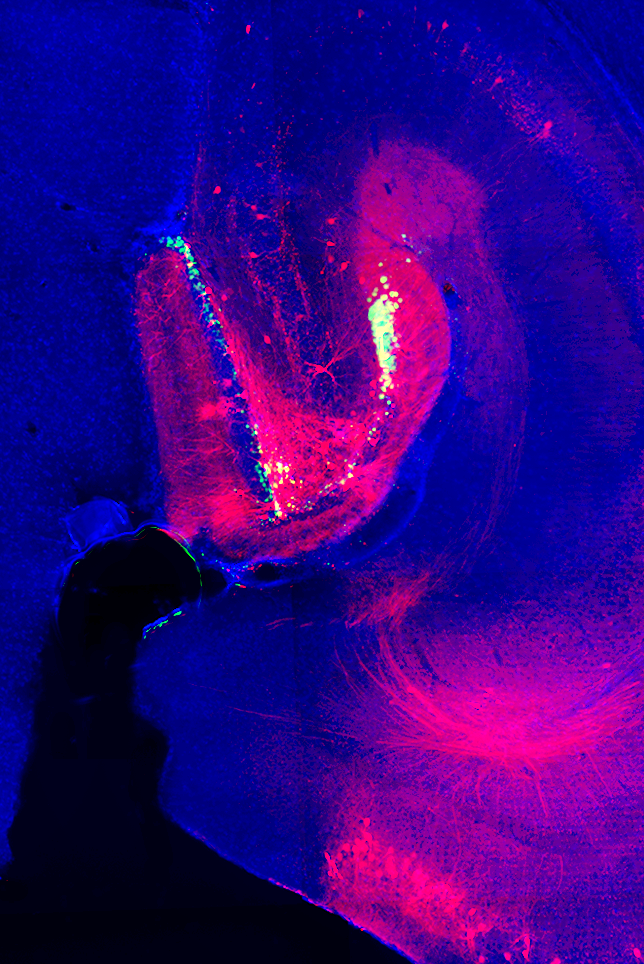

Supplement: Supplementary file 7 — Source data Fig. 4 [file 44321_2024_168_MOESM7_ESM.zip › Figure 4/4A/Nos1-- -2.tif]

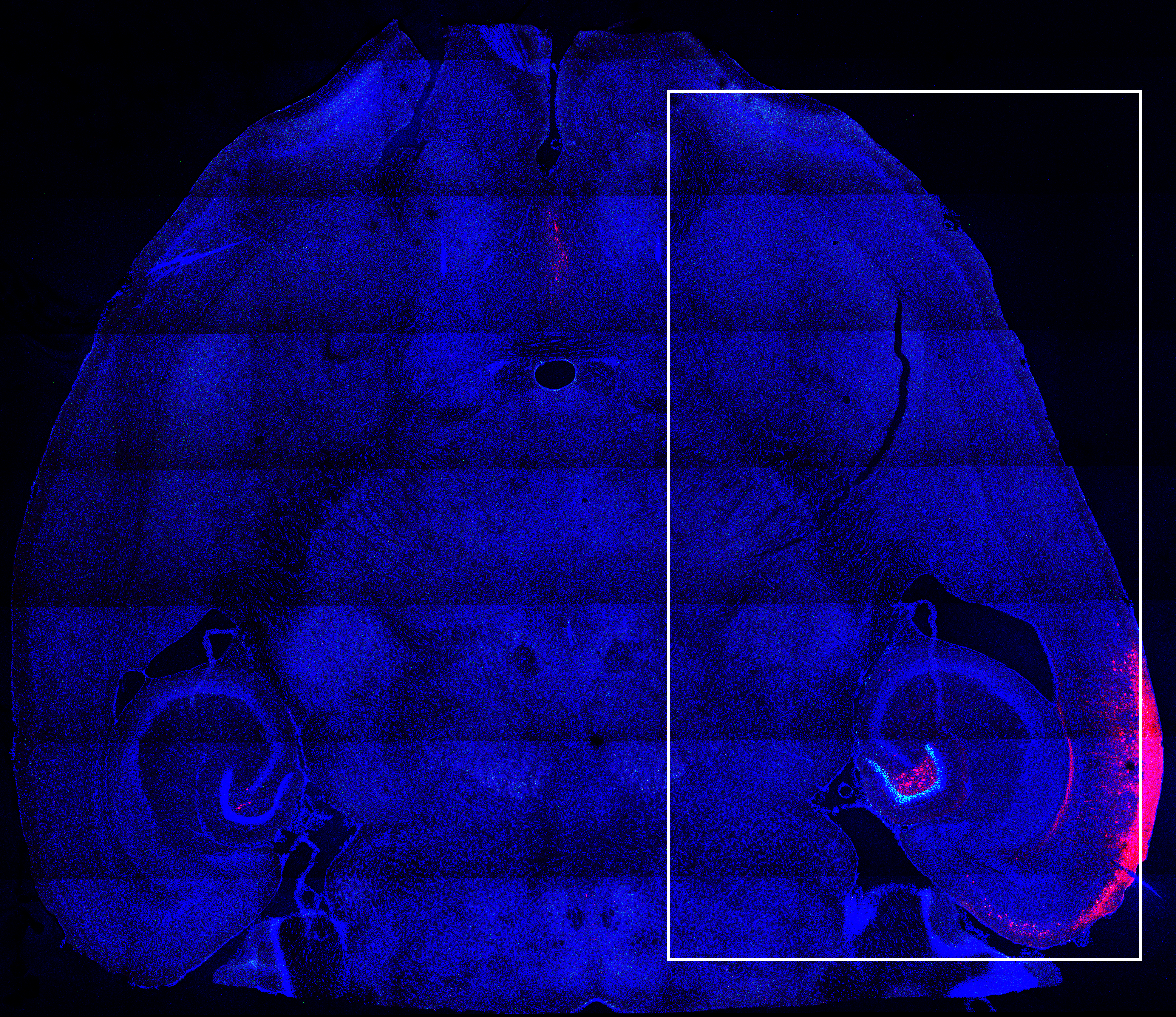

Supplement: Supplementary file 7 — Source data Fig. 4 [file 44321_2024_168_MOESM7_ESM.zip › Figure 4/4A/Nos1-- -3 (original image).tif]

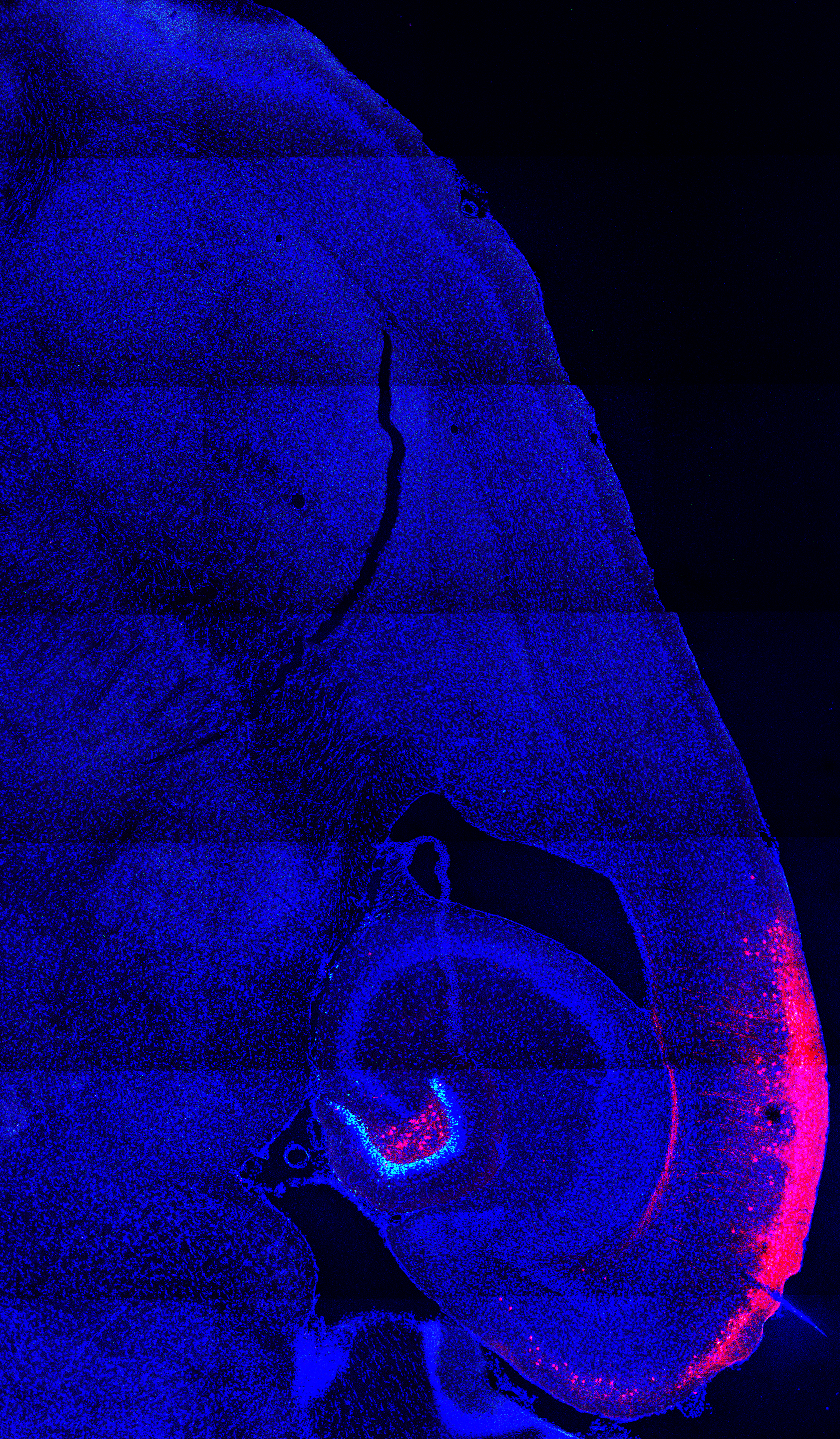

Supplement: Supplementary file 7 — Source data Fig. 4 [file 44321_2024_168_MOESM7_ESM.zip › Figure 4/4A/Nos1-- -3.tif]

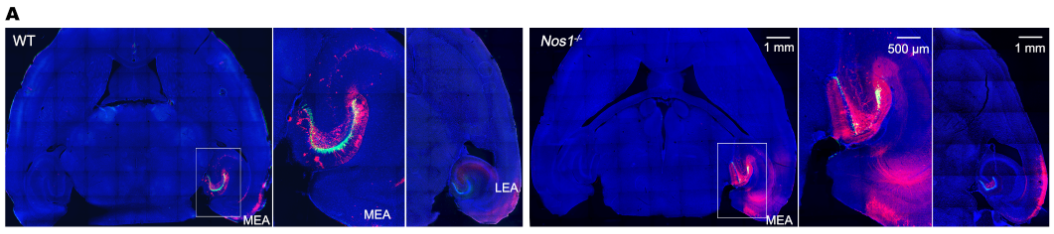


WT-1 WT-2 WT-3 Nos1-- -1 Nos1-- -2 Nos1-- -3

Supplement: Supplementary file 7 — Source data Fig. 4 [file 44321_2024_168_MOESM7_ESM.zip › Figure 4/4A/README.docx]

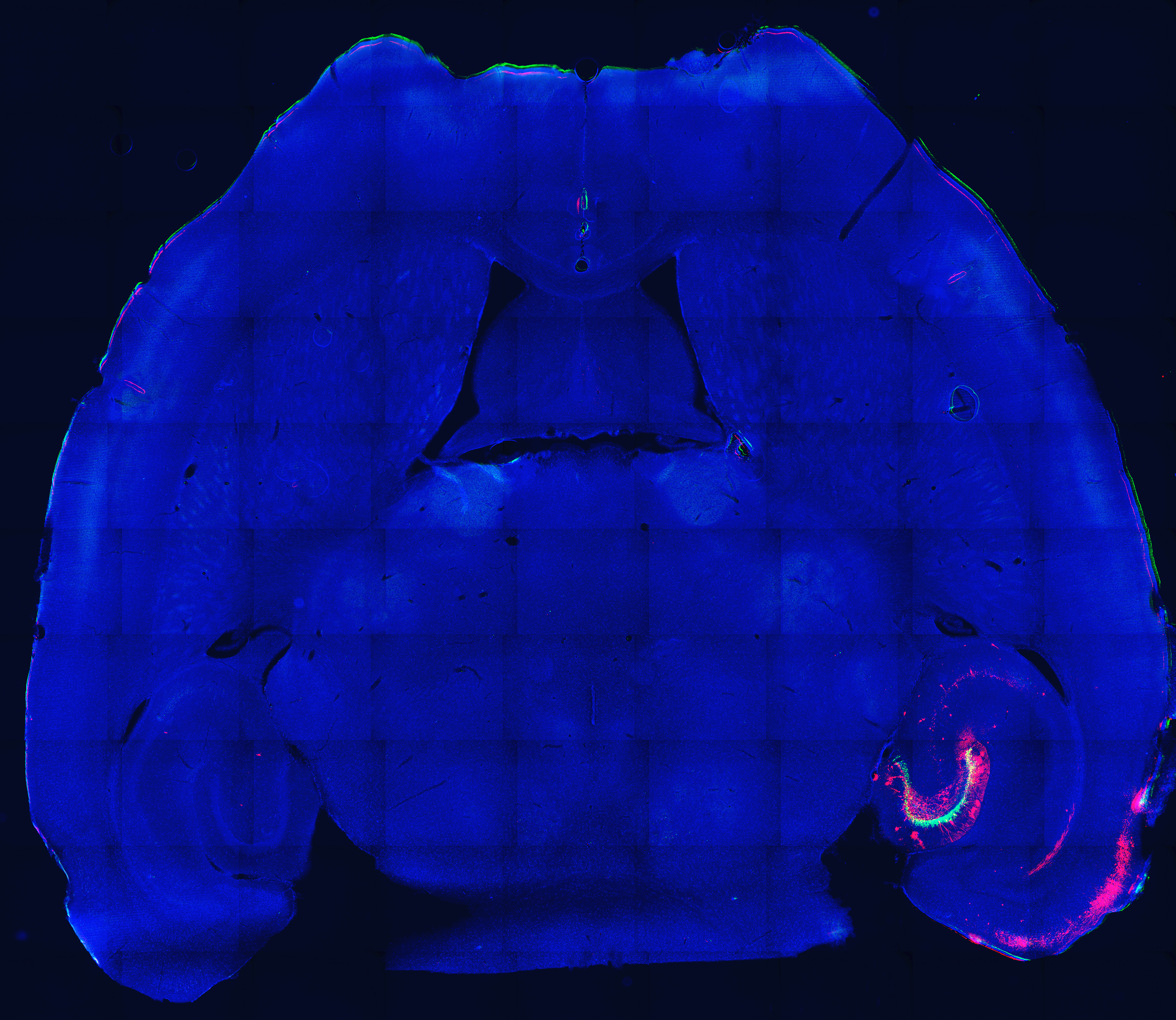

Supplement: Supplementary file 7 — Source data Fig. 4 [file 44321_2024_168_MOESM7_ESM.zip › Figure 4/4A/WT-1.tif]

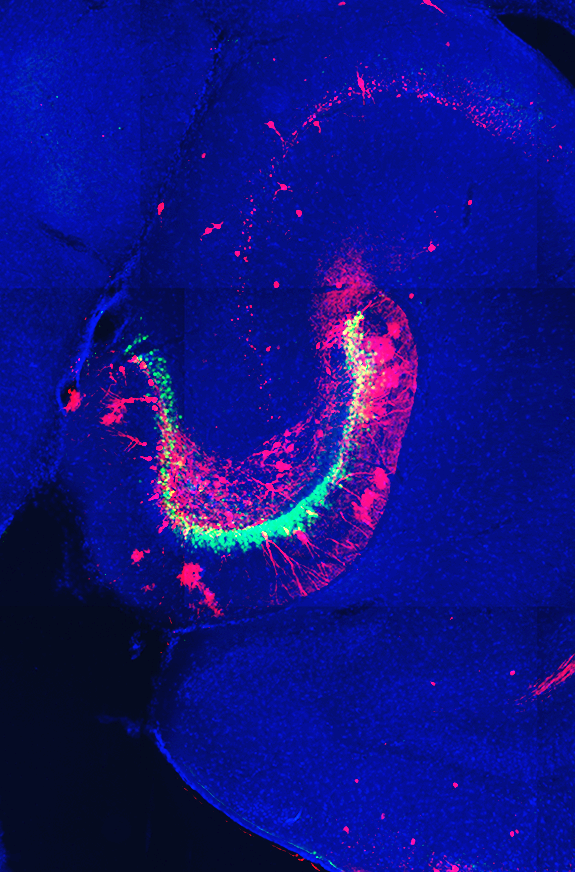

Supplement: Supplementary file 7 — Source data Fig. 4 [file 44321_2024_168_MOESM7_ESM.zip › Figure 4/4A/WT-2.tif]

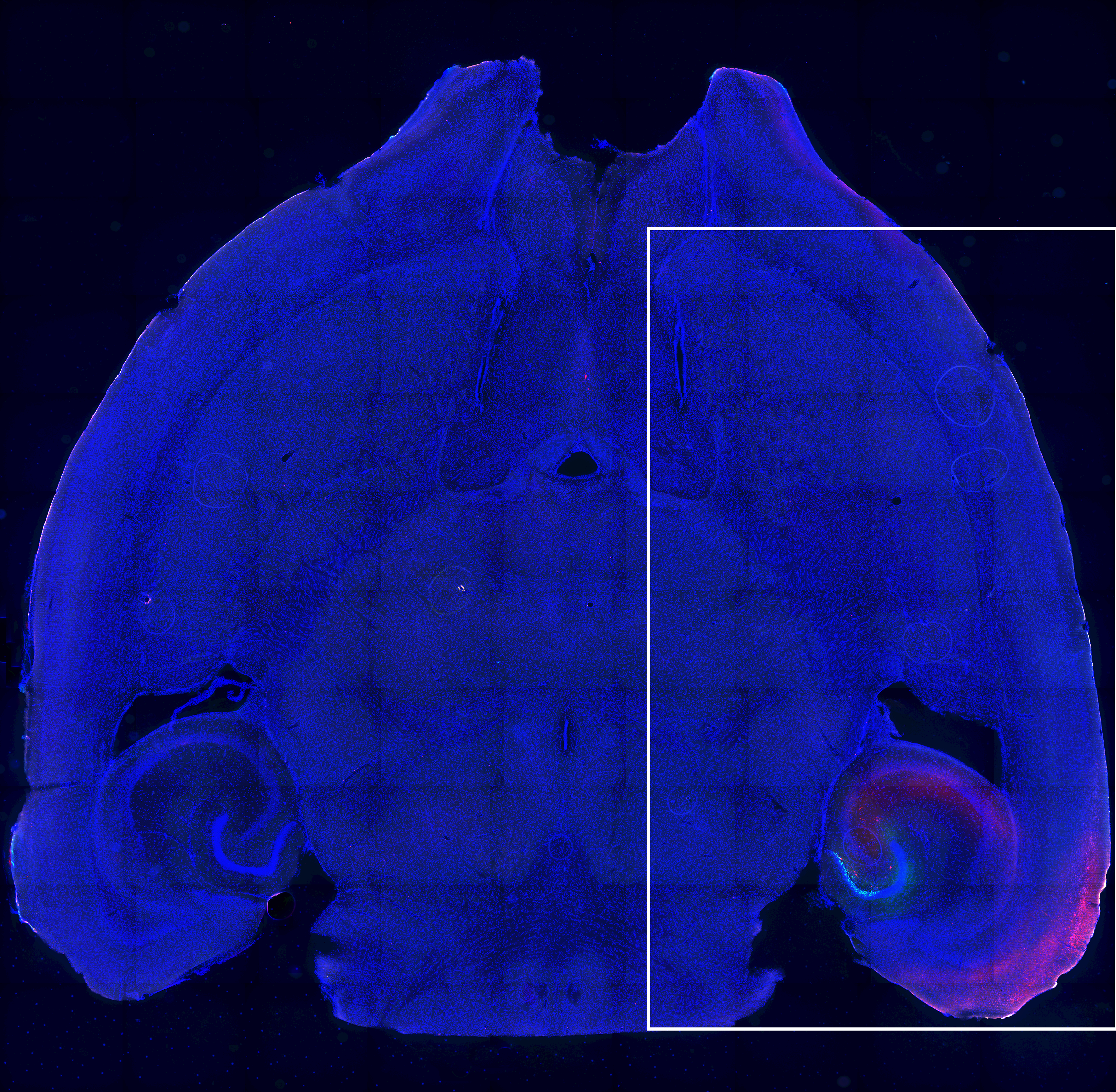

Supplement: Supplementary file 7 — Source data Fig. 4 [file 44321_2024_168_MOESM7_ESM.zip › Figure 4/4A/WT-3 (original image).tif]

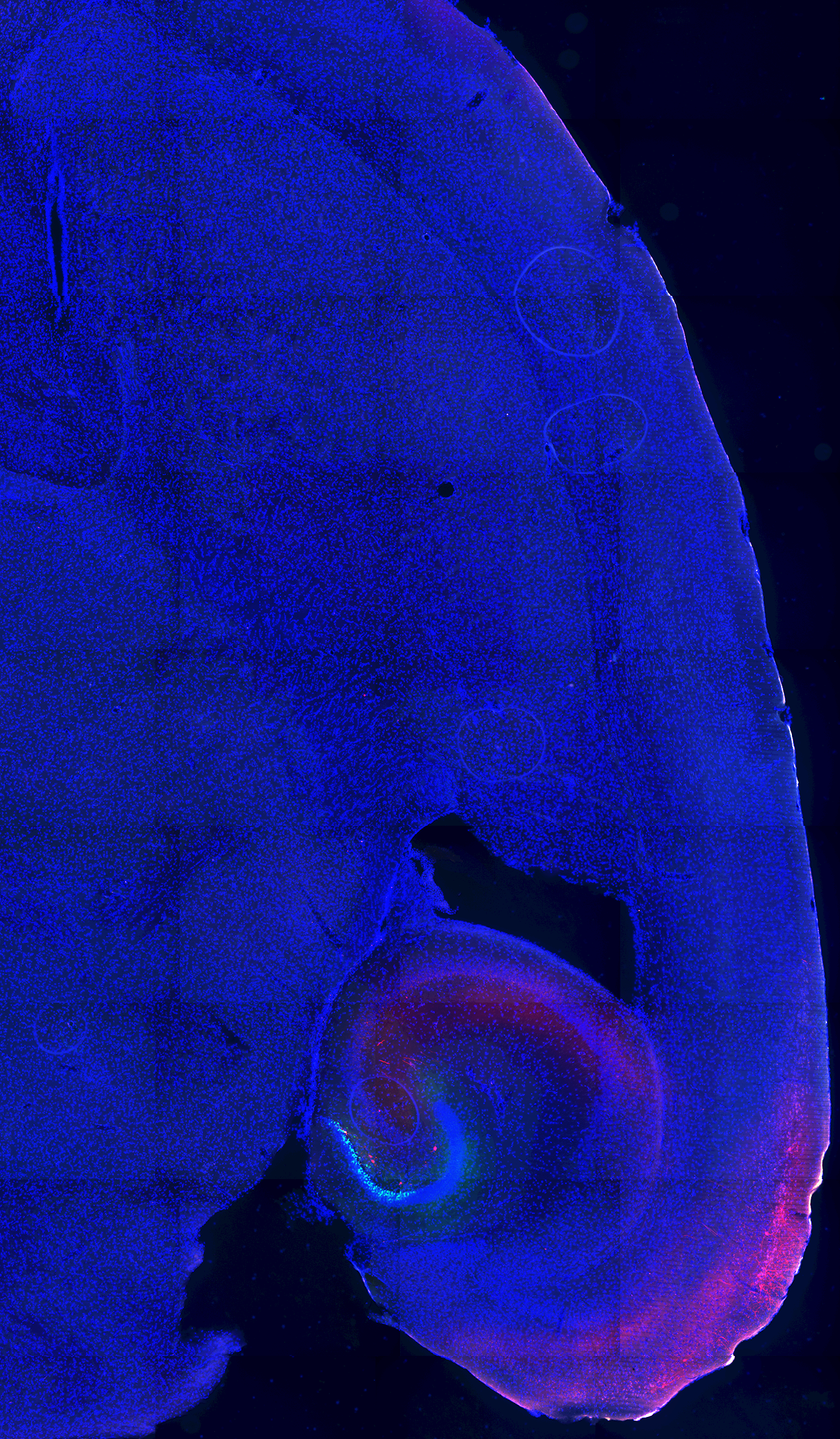

Supplement: Supplementary file 7 — Source data Fig. 4 [file 44321_2024_168_MOESM7_ESM.zip › Figure 4/4A/WT-3.tif]

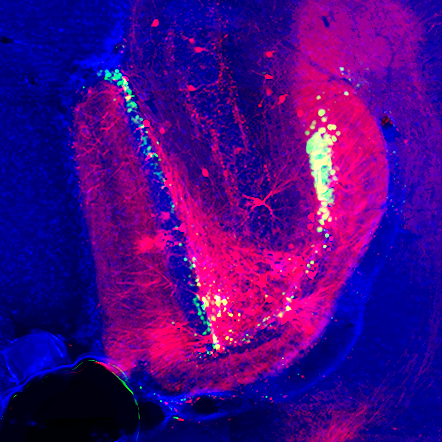

Supplement: Supplementary file 7 — Source data Fig. 4 [file 44321_2024_168_MOESM7_ESM.zip › Figure 4/4B/Nos1--/Merge.tif]

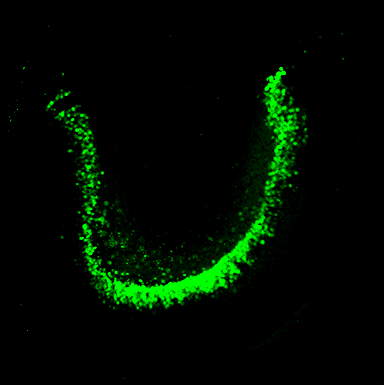

Supplement: Supplementary file 7 — Source data Fig. 4 [file 44321_2024_168_MOESM7_ESM.zip › Figure 4/4B/WT/Green channel.tif]

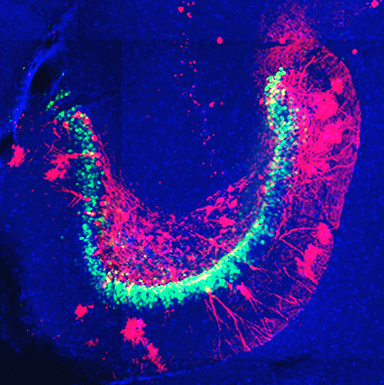

Supplement: Supplementary file 7 — Source data Fig. 4 [file 44321_2024_168_MOESM7_ESM.zip › Figure 4/4B/WT/Merge.tif]

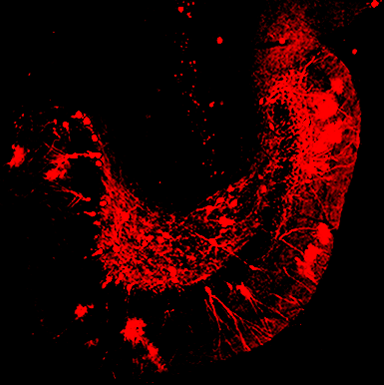

Supplement: Supplementary file 7 — Source data Fig. 4 [file 44321_2024_168_MOESM7_ESM.zip › Figure 4/4B/WT/Red channel.tif]

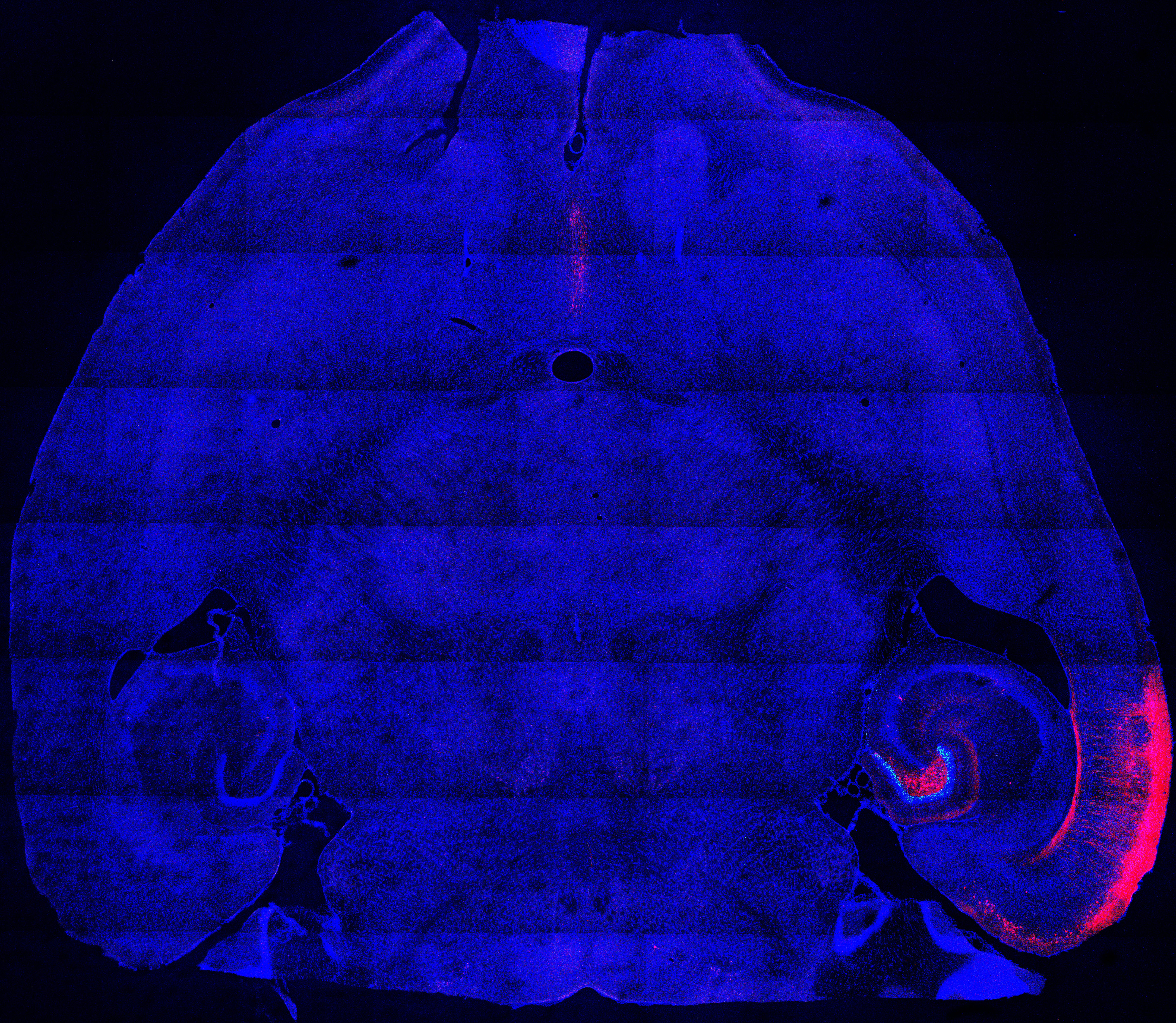

Supplement: Supplementary file 7 — Source data Fig. 4 [file 44321_2024_168_MOESM7_ESM.zip › Figure 4/4E/Nos1_loxp_loxp.tif]

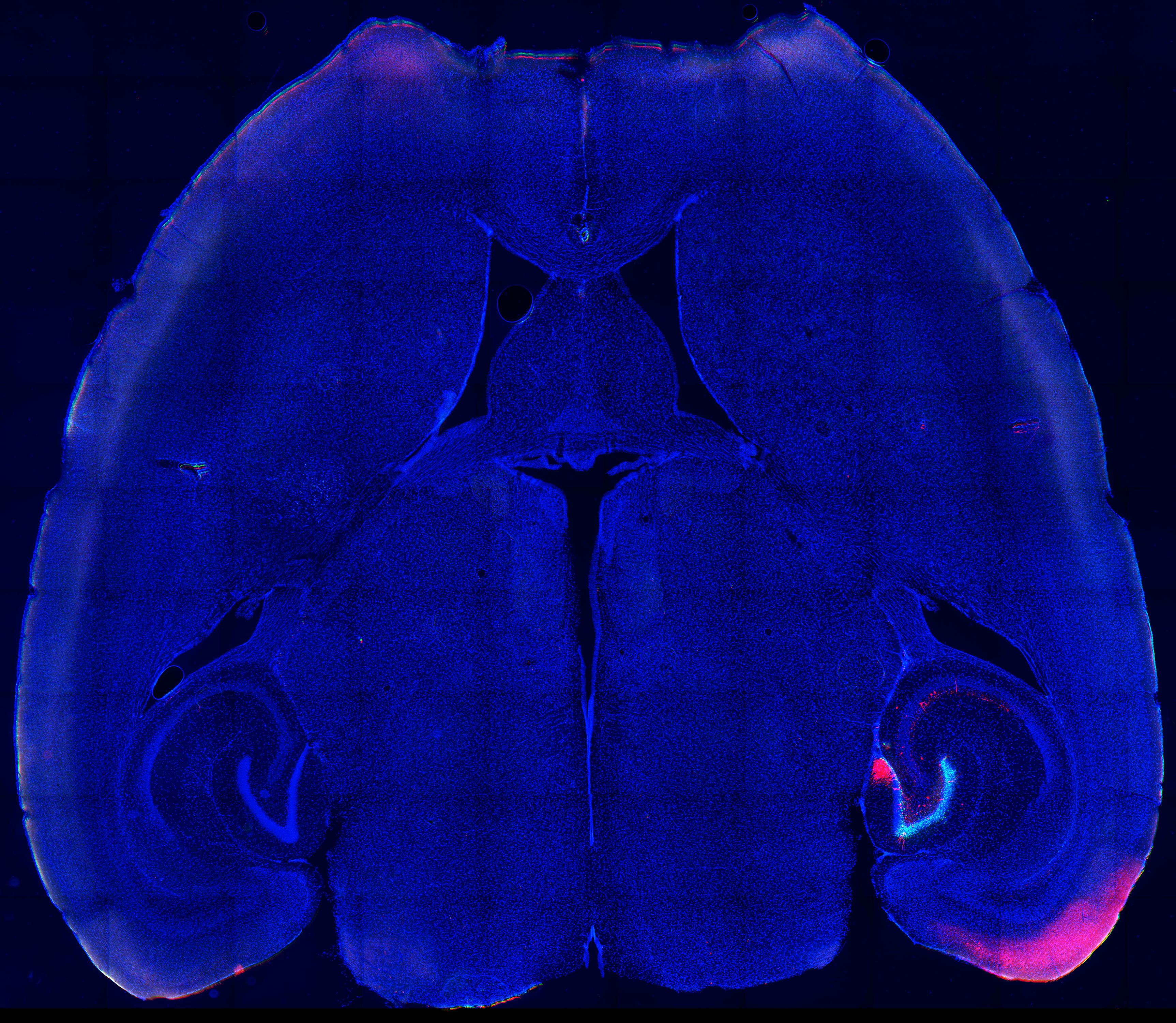

Supplement: Supplementary file 7 — Source data Fig. 4 [file 44321_2024_168_MOESM7_ESM.zip › Figure 4/4E/WT.tif]

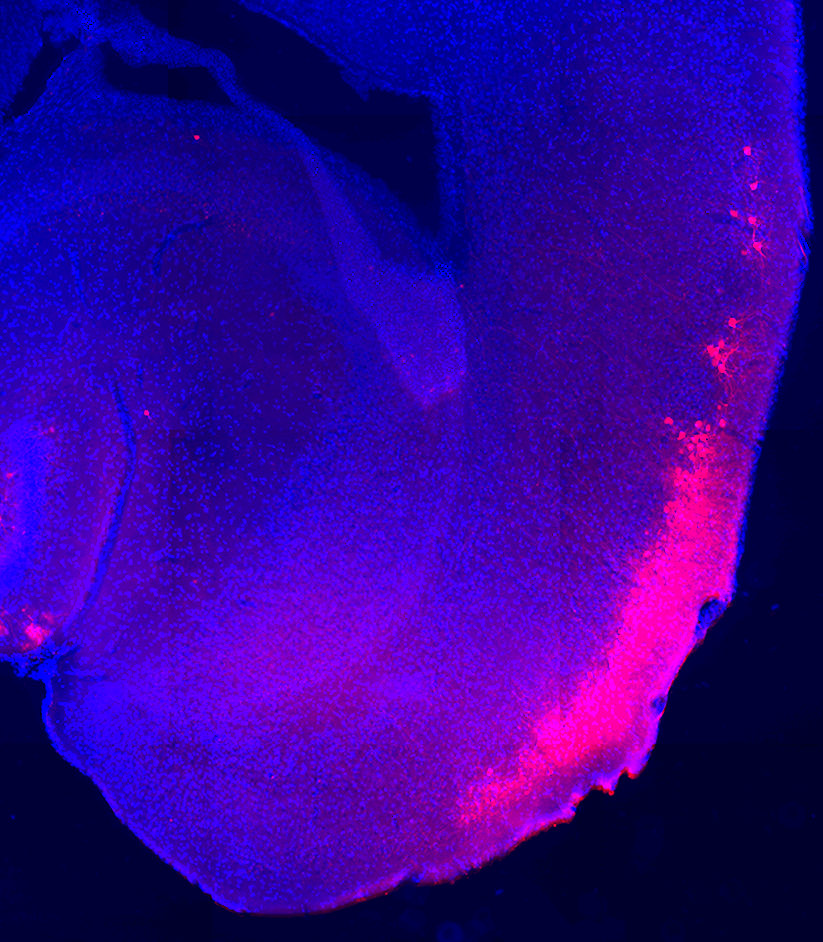

Supplement: Supplementary file 10 — Source data Fig. 7 [file 44321_2024_168_MOESM10_ESM.zip › Figure 7/7B/Control & Vehicle.tif]

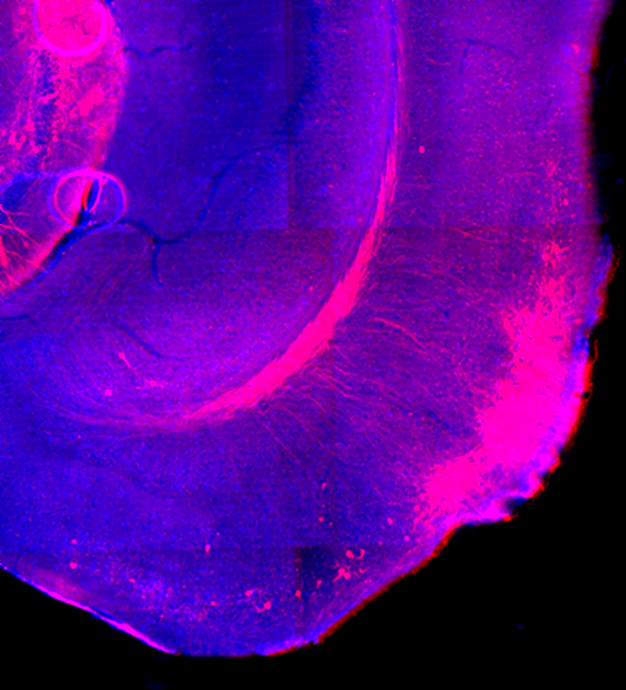

Supplement: Supplementary file 10 — Source data Fig. 7 [file 44321_2024_168_MOESM10_ESM.zip › Figure 7/7B/Pilocarpine & DETA_NONOate.tif]

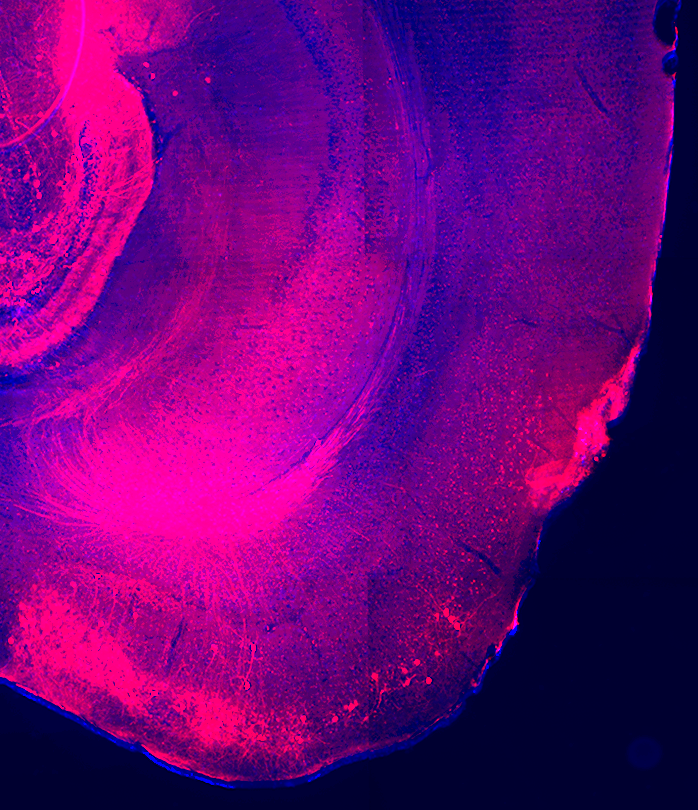

Supplement: Supplementary file 10 — Source data Fig. 7 [file 44321_2024_168_MOESM10_ESM.zip › Figure 7/7B/Pilocarpine & Vehicle.tif]
