# Supplementary material for: A novel anti-epileptogenesis strategy of temporal lobe epilepsy based on nitric oxide donor
Source: EMBO Mol Med. 2024 Dec 9;17(1):85–111. doi: 10.1038/s44321-024-00168-1 (PMC11730642; doi:10.1038/s44321-024-00168-1)
Supplement: Supplementary file 11 — Expanded View Figures [file 44321_2024_168_MOESM11_ESM.pdf]

Expanded View Figures

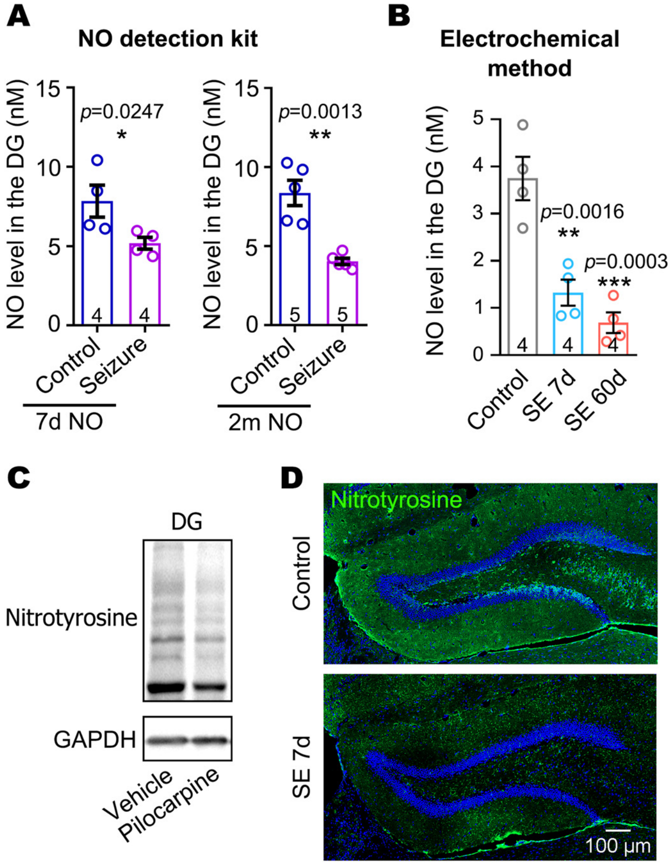

Figure EV1. NO content decreases in TLE mouse models.

(**A**) Data graph showing the concentration of NO in DG 7 days or 2 months after pilocarpine-induced SE using NO detection kit. Students' *t*-test, $n = 4$ mice. (**B**) NO measurements using electrochemical methods. One-way ANOVA, $n = 5$ mice. (**C**) Representative images showing decreased nitrotyosine-modified proteins in the DG 2 months after pilocarpine administration. The same result was observed in 5 mice. (**D**) Representative photos of immunofluorescence of nitrotyosine in the DG 7 days after pilocarpine-induced SE. (**E**) Representative image and data graph showing nNOS expression in DG 14 days after KA-induced SE. Students' *t*-test, $n = 7$–9 mice. (**F**) Representative image and data graph showing nNOS expression in DG 28 days after PTZ-induced SE. Students' *t*-test, $n = 4$–10 mice. Error bars correspond to ± s.e.m. $^{*}P < 0.05$, $^{**}P < 0.01$, $^{***}P < 0.001$, $^{***}P < 0.001$, $^{****}P < 0.0001$.

© The Author(s)

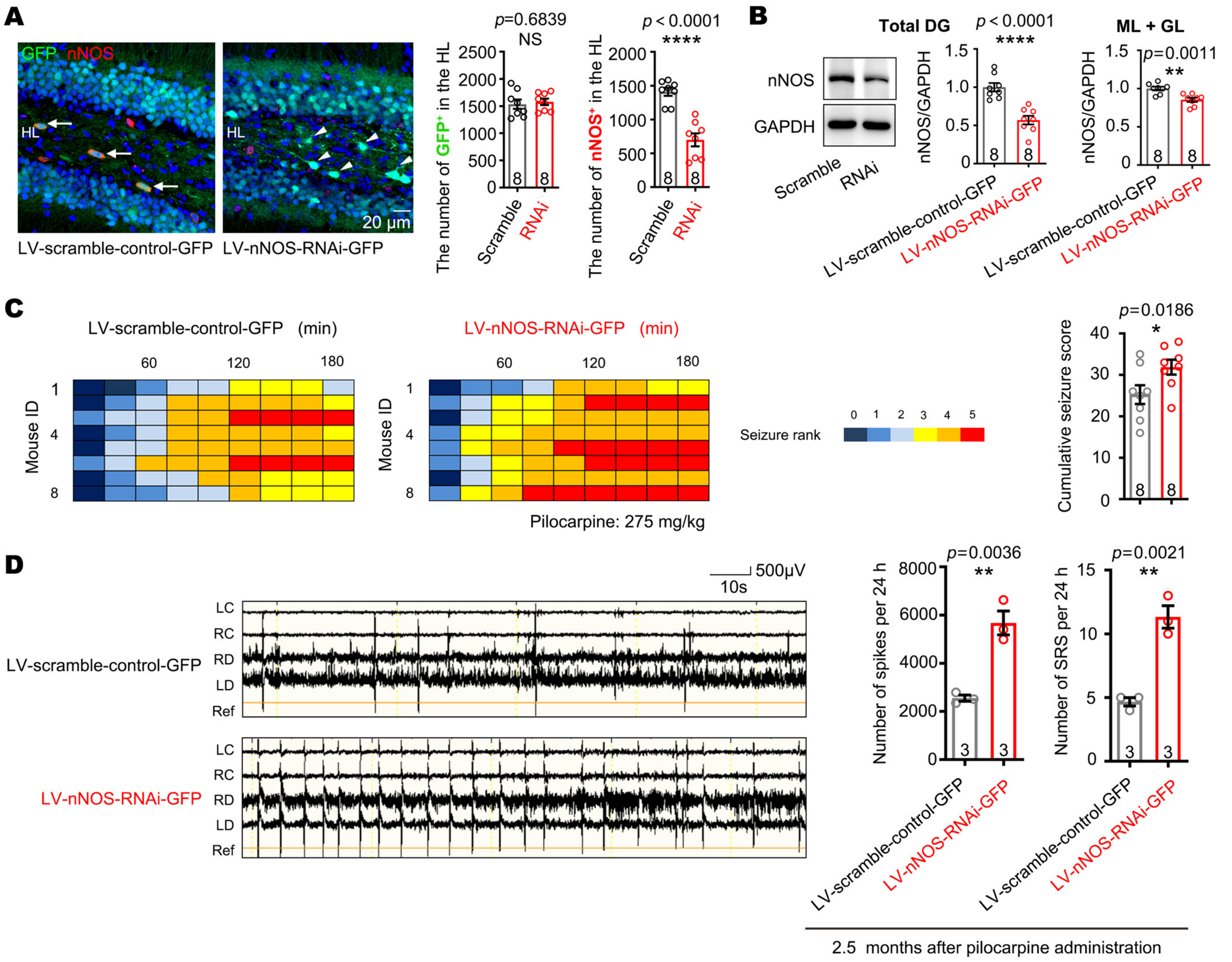

Figure EV2. Knockdown of nNOS increases susceptibility of inducing status epilepticus and aggravates epileptogenesis in the pilocarpine mouse model of TLE.

(**A**) Representative photos and data graphs of nNOS-positive/GFP-positive cells in the hilus 1 month after injection 1 μL of LV-scramble-control-GFP or LV-nNOS-RNAi-GFP into the hilus. Notably, less $nNOS^+$ cells were observed after injection of LV-nNOS-RNAi-GFP. Arrows indicated transduced hilar nNOS-positive interneurons and arrowheads indicated transduced hilar nNOS-negative interneurons. The same observation was repeated in 5 mice. Student's *t*-test, $n = 8$ mice. (**B**) Western blot showing deceased content of nNOS protein in the DG and in the ML + GL regions 1 month after injection 1 μL of LV-nNOS-RNAi-GFP into the hilus. Student's *t*-test, $n = 8$ mice. (**C**) Heat map and data graph showing cumulative seizure score for 3 h after pilocarpine treatment at a dose of 275 mg/kg in mice. The mice received injection 1 μL of LV-scramble-control-GFP or LV-nNOS-RNAi-GFP into the hilus 1 month before pilocarpine administration. Student's *t*-test, $n = 12$ mice. (**D**) Representative images and data graphs of EEG recordings showing brain waves of mice 2.5 months after pilocarpine-induced SE. The mice received injection 1 μL of LV-scramble-control-GFP or LV-nNOS-RNAi-GFP into the hilus 1 month before pilocapine administration. Student's *t*-test, $n = 3$ mice. GL granule layer, ML molecular layer, HL hilus, LC left cortex, RC right cortex, RD right DG, LD left DG, Ref Reference. Error bars correspond to ± s.e.m. $*P < 0.05$, $**P < 0.01$, $****P < 0.0001$, $****P < 0.0001$, *NS*, not significant.

 © The Author(s)

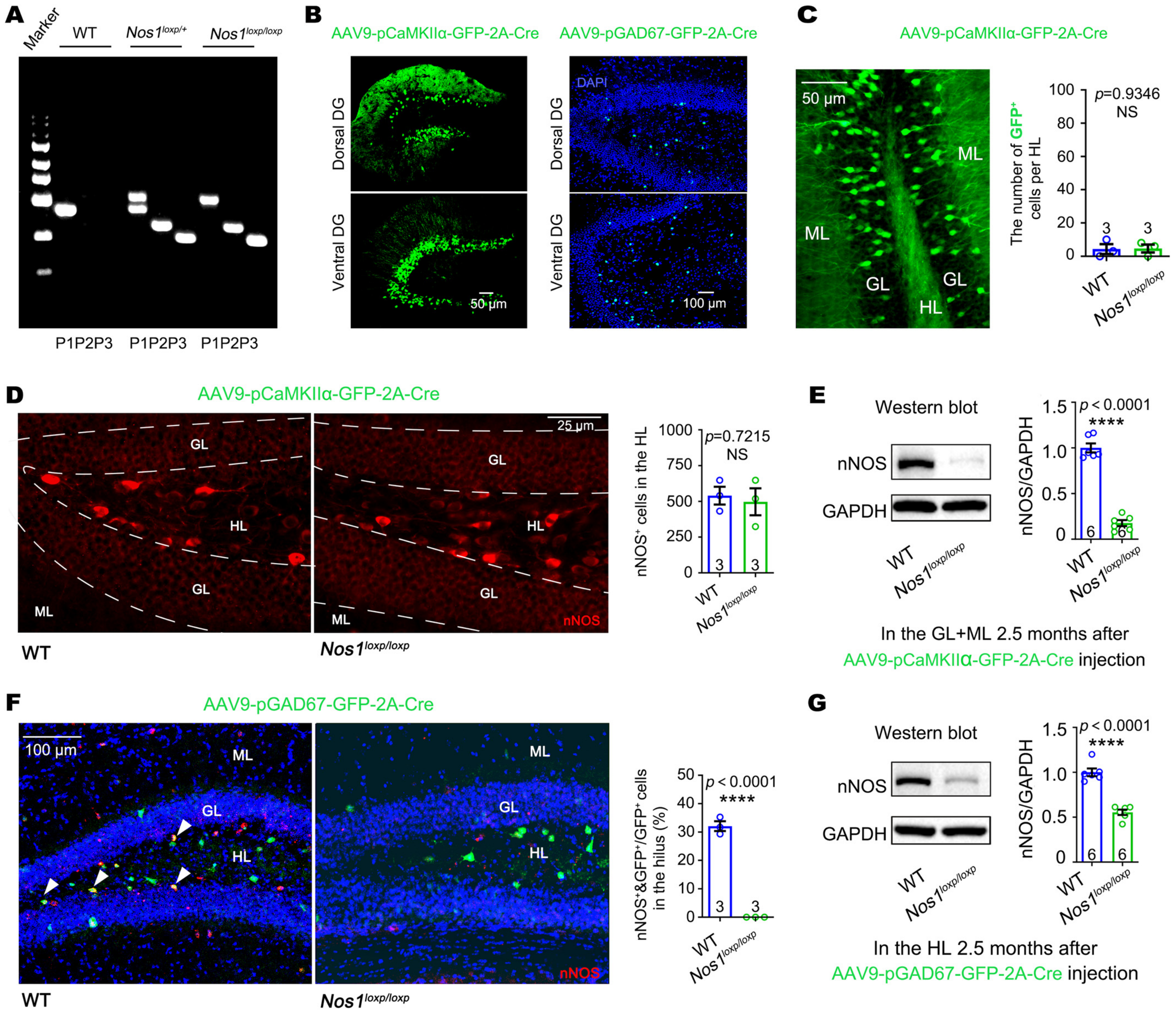

Figure EV3. Increased susceptibility to SE induction after deletion of nNOS from hilar interneurons but not from DGCs.

(**A**) Representative genotyping of $Nos1^{loxp/loxp}$ mice. P1, P2, and P3 showed productions synthesized by three primers, respectively. (**B**) The distribution of AAV9-pCaMKIIα-GFP-2A-Cre virus or AAV9-pGAD67-GFP-2A-Cre virus transduced neurons along the DG dorsal-ventral axis. The same pattern was observed in all injected mice. (**C**) Representative photo of AAV9-pCaMKII-GFP-2A-Cre transduction and data graph showing the number of GFP$^+$ cells in the hilus were not altered. Student's *t*-test, $n = 3$ mice. (**D**) Representative photos and data graph showing nNOS-positive cells in the hilus 1 month after injection of AAV9-pCaMKIIα-GFP-2A-Cre into the hilus of WT or $Nos1^{loxp/loxp}$ mice. No significant difference was observed. Student's *t*-test, $n = 3$ mice. (**E**) The expression of nNOS protein in the granule layer and molecular layer were reduced after injection of AAV9-pCaMKIIα-GFP-2A-Cre virus into the hilus of $Nos1^{loxp/loxp}$ mice. Student's *t*-test, $n = 6$ mice. (**F**) Representative photos and data graphs showed that the number of nNOS$^+$ cells in the hilus was reduced and no granule neurons were transduced. Arrowheads indicated transduced hilar nNOS$^+$ neurons. Student's *t*-test, $n = 3$ mice. (**G**) The expression of nNOS protein in the hilus was reduced after injection of AAV9-pGAD67-GFP-2A-Cre virus into the hilus of $Nos1^{loxp/loxp}$ mice. Student's *t*-test, $n = 6$ mice. GL granule layer, ML molecular layer, HL hilus. Error bars correspond to ± s.e.m. ****$P < 0.0001$, *NS*, not significant.

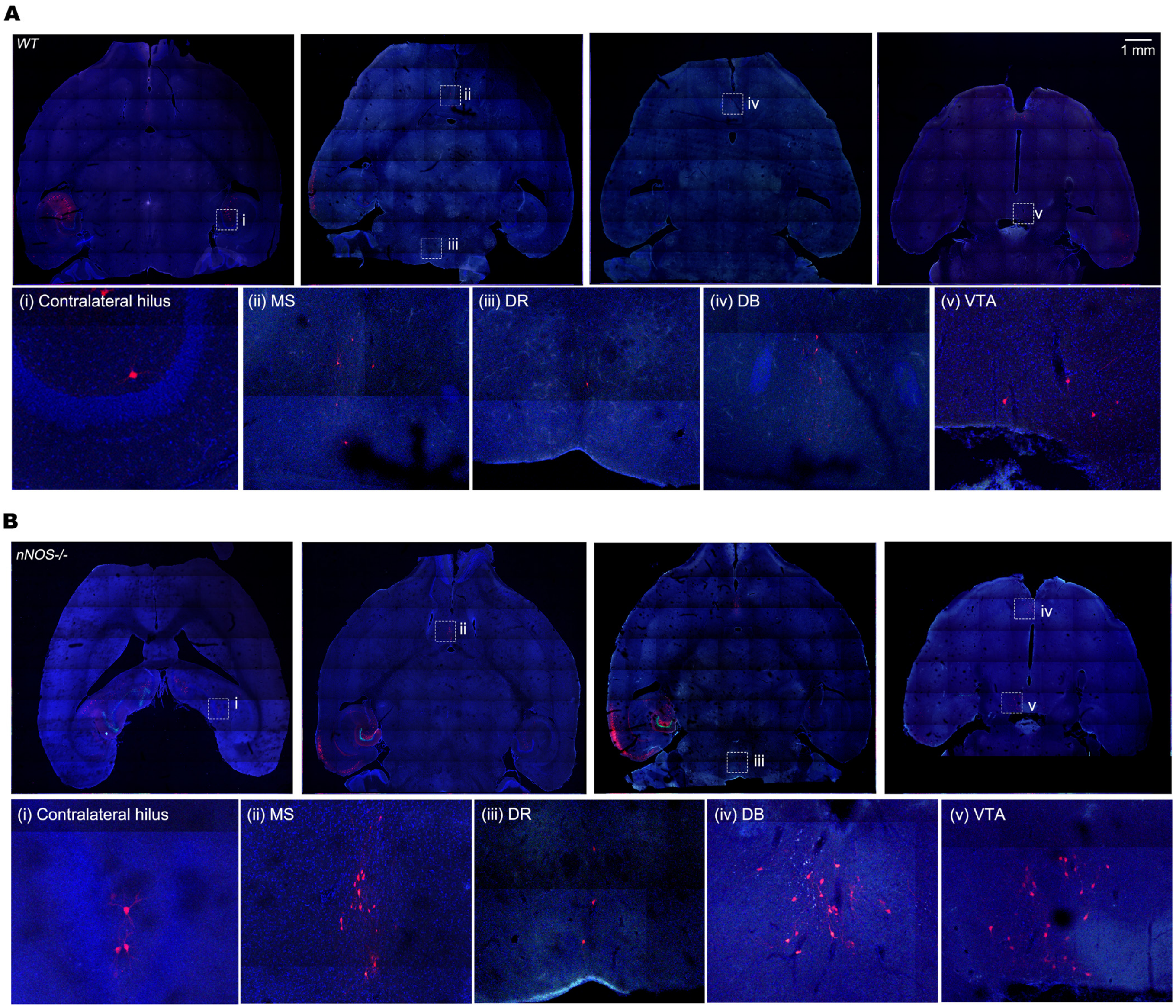

Figure EV4. Distribution of traced cells projecting to DGCs.

(**A**) Representative photos showing the distribution of traced cells projecting to DGCs in the brain of WT mice. (**B**) Representative photos showing the distribution of traced cells projecting to DGCs in the brain of *Nos1*$^{-/-}$ mice. (i–v) were zoomed photos from white squares, respectively. MS medial septum, DR dorsal raphe, DB diagonal band, VTA ventral tegmental area.

 © The Author(s)

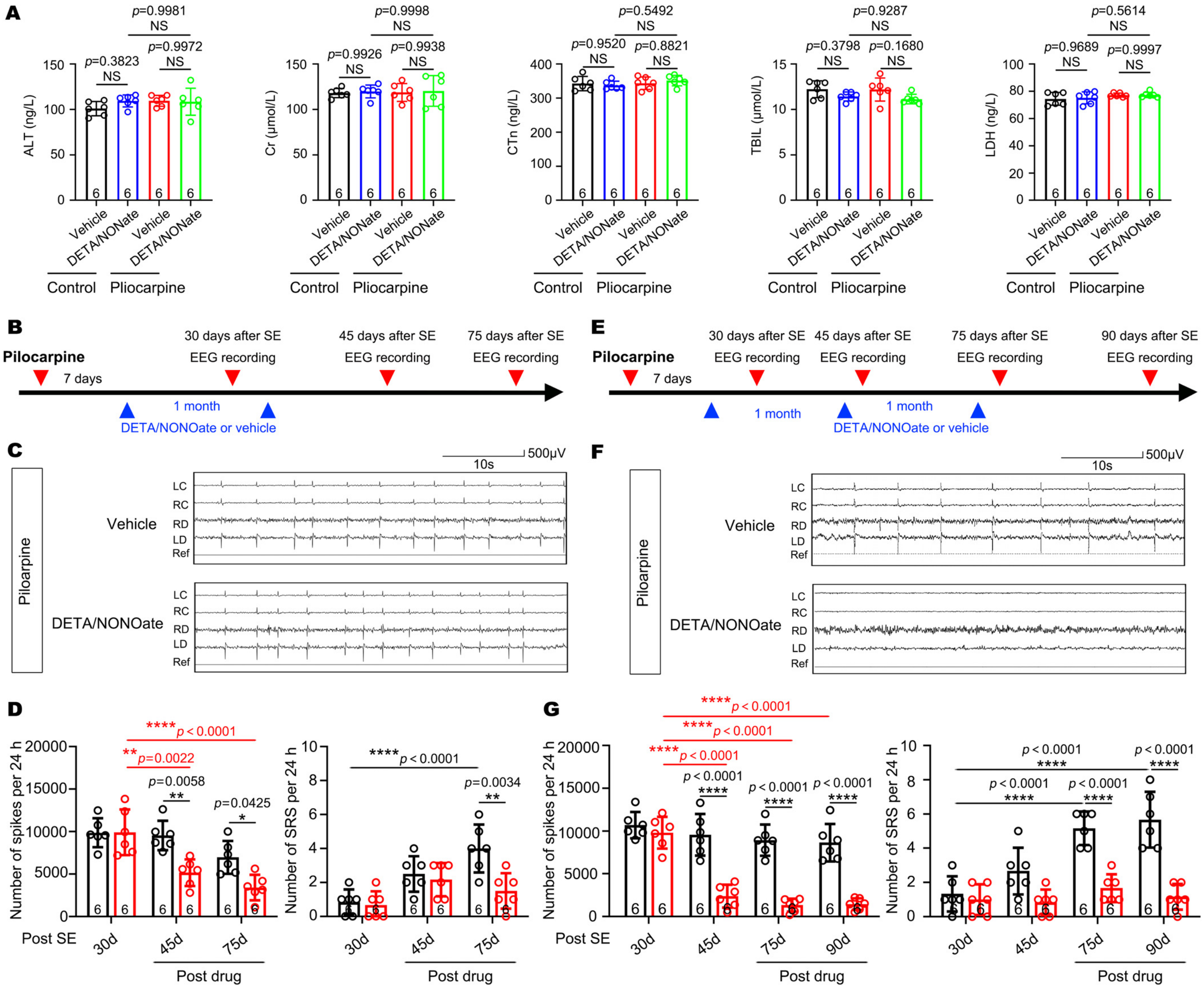

Figure EV5. Chronic treatment with NO Donor prevents epileptogenesis with long-lasting effect.

(**A**) Quantification of plasmatic ALT, TBIL, Cr, LDH, and CTn using ELISA kits. Two-way ANOVA, n- = 6 mice. (**B–D**) Experimental paradigm (**B**), representative EEG recordings (**C**), and data graphs (**D**), showing chronic treatment with DETA/NONOate (1 mg/kg, i.p., 1 time per day) for 1 month after pilocarpine-induced SE for 7 days prevented the development of SPKs and SRS. Two-way ANOVA, $n = 6$ mice. (**E–G**) Experimental paradigm (**E**), representative EEG recordings (**F**), and data graphs (**G**), showing chronic treatment with DETA/NONOate (1 mg/kg, i.p., 1 time per day) for 1 month after pilocarpine-induced SE for 37 days prevented the development of SPKs and SRS. Two-way ANOVA, $n = 6$ mice. ALT alanine aminotransferase, TBIL total bilirubin, Cr creatinine, LDH lactate dehydrogenase, CTn cardiac troponin. Error bars correspond to ± s.e.m. *$P < 0.05$, **$P < 0.01$, ****$P < 0.0001$, *NS*, not significant.

© The Author(s)
